# Shallow seamounts are “oases” and activity hubs for pelagic predators in a large-scale marine reserve

**DOI:** 10.1371/journal.pbio.3003016

**Published:** 2025-02-04

**Authors:** Sam B. Weber, Andrew J. Richardson, Christopher D. H. Thompson, Judith Brown, Fabio Campanella, Brendan J. Godley, Nigel E. Hussey, Jessica J. Meeuwig, Paul Rose, Nicola Weber, Matthew J. Witt, Annette C. Broderick

**Affiliations:** 1 Centre for Ecology and Conservation, University of Exeter, Cornwall Campus, Penryn, Cornwall, United Kingdom; 2 Ascension Island Government Conservation & Fisheries Department, Georgetown, Ascension Island; 3 Marine Futures Lab, School of Biological Sciences, University of Western Australia, Crawley, Australia; 4 National Geographic Pristine Seas, Washington DC, United States of America; 5 Centre for Fisheries and Aquaculture Science, Lowestoft, Suffolk, United Kingdom; 6 National Research Council (CNR), Institute for Biological Resources and Marine Biotechnologies (IRBIM), Ancona, Italy; 7 Environment and Sustainability Institute, University of Exeter, Cornwall Campus, Penryn, Cornwall, United Kingdom; 8 University of Windsor—Integrative Biology, Windsor, Ontario, Canada; 9 Faculty of Health and Life Sciences, University of Exeter, Exeter, United Kingdom; University of California, UNITED STATES OF AMERICA

## Abstract

Seamounts have been likened to “oases” of life in the comparative deserts of the open ocean, often harbouring high densities of threatened and exploited pelagic top predators. However, few such aggregations have been studied in any detail and the mechanisms that sustain them are poorly understood. Here, we present the findings of an integrated study of 3 previously unexplored seamounts in the tropical Atlantic, which aimed to investigate their significance as predator “hotspots” and inform their inclusion in one of world’s largest marine reserves. Baited underwater video and visual census transects revealed enhanced diversity and biomass of pelagic top predators, including elevated abundances of 7 species of sharks, predatory fish, and seabirds, within 5 km of 2 shallow seamounts (<100 m), but not a third deeper seamount (260 m). Hydroacoustic biomass of low- and mid-trophic level “prey” was also significantly elevated within 2.5 km of shallow seamounts. However, we found no evidence of enhanced primary productivity over any feature, suggesting high faunal biomass is sustained by exogenous energy inputs. Relative biomass enrichment also increased with trophic level, ranging from a 2-fold increase for zooplankton to a 41-fold increase for sharks. Tracking of the dominant predator species revealed that individual sharks (Galapagos, silky) and tuna (yellowfin, bigeye) often resided around seamounts for months to years, with evidence of connectivity between features, and (in the case of sharks) were spatially aggregated in localised hotspots that coincided with areas of high mid-trophic biomass. However, tuna and silky sharks also appeared to use seamounts as “hubs” in more extensive pelagic foraging ranges, which may help explain disproportionately high predator density. Our results reinforce the conservation significance of shallow seamounts for many marine top predators and offer fundamental insights into their functional roles as both prey “oases” and activity hubs for these species.

## Abstract 2

Seamounts have been likened to ‘oases’ of life in the comparative deserts of the open ocean. This integrated study of tropical seamounts suggests that high faunal biomass is sustained by exogenous energy inputs and that they act as aggregating ’hubs’ for marine top predators.

## Introduction

Seamounts have long been recognised as biodiversity “hotspots” in the comparatively featureless expanses of the open ocean, often harbouring high faunal biomass [1] and significantly elevated abundance and diversity of pelagic top predators (e.g., sharks, seabirds, billfish, and tunas) compared to surrounding oceanic habitats [2–4]. The predictable concentration of large, predatory species found around many seamounts has made them targets for fisheries [1,5] and priorities for area-based conservation measures [6], as well as offering fundamental insights into the processes that shape the distribution of life in our oceans. However, despite growing awareness of their ecological significance, most seamounts remain unstudied and unprotected. According to some recent estimates, of the ca. 38,000 seamounts identified worldwide [7,8], fewer than 2% are contained within existing marine protected area (MPA) networks [7] and <4% have been explored for scientific purposes, with much of what is known based on a few well-studied examples [9]. Although detailed studies of seamount predator assemblages are limited, 2 general conclusions that have emerged are: (1) that not all seamounts are equally important for pelagic predators [10]; and (2) that not all predator species interact with seamounts in the same way [10]. At a time when many marine top predators are declining globally due to human activities [11,12], there is a pressing need to identify and protect the most important seamount predator hotspots and explore the underlying mechanisms that sustain them.

Two broad sets of mechanisms have been proposed to explain the predator aggregating effect of seamounts, which we refer to as the “oasis hypothesis” and the “hub hypothesis.” According to the oasis hypothesis, unique biophysical interactions around seamounts create areas of consistently high biological production that are capable of sustaining elevated biomass across trophic levels [13]. This trophic enrichment may occur in 2 ways. First, seamount topography may disrupt the flow of ocean currents and tides causing deeper, nutrient rich, waters to be uplifted into surface layers [14]. This influx of limiting nutrients fuels phytoplankton growth and triggers a cascade of biological productivity, creating a fertile “oasis” that attracts large predators (“seamount-induced chlorophyll enrichment”; SICE) [14,15]. Support for SICE has so far been mixed, with only 27% of shallow seamounts globally shown to influence local primary productivity, suggesting it is a site-specific phenomenon [15]. Alternatively, seamounts may boost productivity by concentrating planktonic prey from exogenous sources: a process known as “trophic focussing” [16]. This can occur where shallow seamount topography blocks the daily vertical migrations of zooplankton, trapping them on the seabed where they are accessible to meso-predators (“DVM trapping”) [16]. Rocky seamount substrates may also provide habitat for benthic filter feeders and site-attached demersal fish that extract suspended food from accelerated bottom flows and “fix” energy on the seamount [16]. These trophic subsidies are hypothesised to flow through food webs to sustain increased abundance of top predators, although few studies have measured biomass accumulation on seamounts across trophic levels.

In addition to concentrating prey, seamounts may also act as assembly areas or “hubs” for non-foraging related activities. As isolated landmarks in featureless oceanic expanses, seamounts may act as navigational waypoints for mobile predators on extended migrations [17], or as meeting points for important ecological functions and life history events such as mating [18] or cleaning [19]. Predator tracking studies also suggest that seamounts may serve as social refuges for pelagic central-place foragers that disperse into surrounding open ocean habitats to feed [20,21]. Seamount predator aggregations may therefore comprise of a mixture of resident populations and transient or recurrent visitors [22,23] and extend well beyond the summits themselves [2]. These observations have important implications both for the effectiveness of area-based conservation measures and for the spatial scale of protection needed. However, comparatively few studies have investigated the residence times and spatial ecology of mobile top predators around seamounts in any detail.

**Fig 1:**
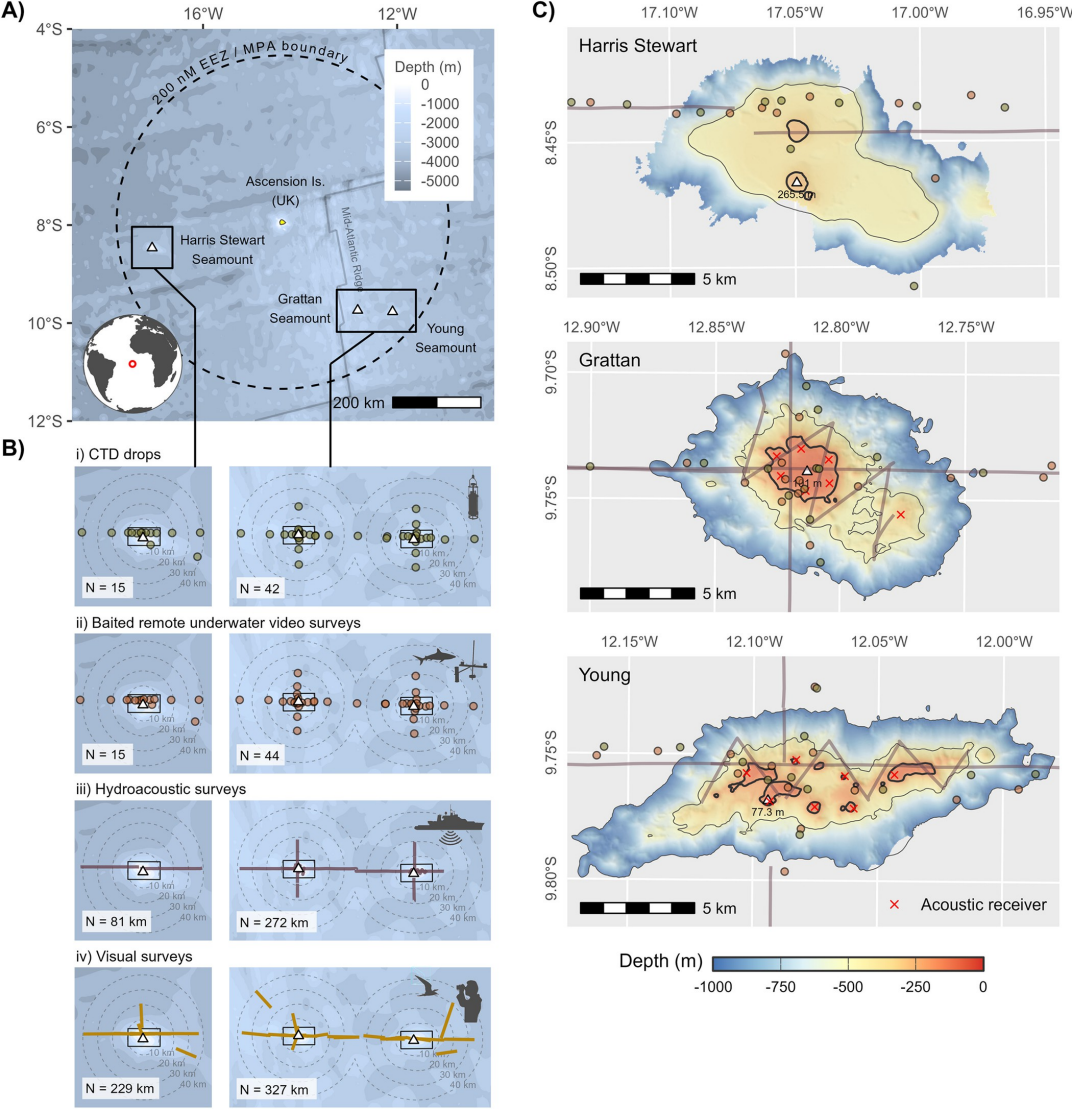
Study location and design. ** (A)** Geographic locations (triangles) of the Harris Stewart, Grattan and Young seamounts within the 200 nautical mile EEZ of Ascension Island, designated in 2019 as a large-scale MPA. **(B)** Sampling sites (points; i, ii) and survey transects (lines; iii, iv) used to assess the “biodiversity footprint” of the study seamounts, along with their respective sample sizes (N). Distance buffers calculated from the 300 m isobath (Harris Stewart Seamount) or 200 m isobath (Southern Seamounts) and are also overlain for reference. **(C)** Expanded views of the bathymetry and sampling locations around the summit of each feature (boxes shown in (B)) based on multibeam data (Simrad EM120) gathered during the study. The depth contour used to define the “summit” of each feature for examining seamount distance effects is marked in bold (200 m for Southern Seamounts and 300 m for Harris Stewart). The data underlying this figure can be found in S1 Data. Bathymetric basemaps and land vector data in A and B were sourced from GEBCO and Natural Earth, respectively. EEZ, exclusive economic zone; MPA, marine protected area.

To better understand the predator-aggregating effect of seamounts, integrated studies are needed that consider biological enrichment across trophic levels and characterise predator assemblages at different levels of organisation, from individuals to communities. Here, we report on the findings of such an assessment that focussed on 3 tropical Atlantic seamounts located in the exclusive economic zone (EEZ) of Ascension Island (UK). The study was motivated by the need to inform the designation of a large-scale MPA and aimed to establish the significance of the focal seamounts for pelagic predators and explore the biophysical mechanisms that give rise to them. Specifically, we ask: (1) Do Ascension Island’s seamounts support higher abundance and diversity of pelagic predators than surrounding open-ocean habitats, and how spatially extensive are such aggregations? (2) Can predator aggregations be explained by an “oasis effect” linked to locally enhanced productivity or prey availability? (3) How do individual pelagic predators interact with seamounts over different timescales and how extensive are their foraging ranges? To address these questions, a suite of complementary marine survey techniques were used to characterise ecological gradients along transects radiating from the summit of each feature to a distance of approximately 40 km (the limit of the “seamount effect” reported by Morato and colleagues [2]) (Fig 1). Focussed tagging of the key predator species observed in these surveys was then used to gather individual-level data on residency and movements around seamounts over longer timescales. With few exceptions, all sampling was carried out during a single expedition, giving a rare, contemporaneous view of the bio-aggregating effect of seamounts across multiple trophic and organisational levels.

## Results

### The Seamounts

The study explored all major known seamounts located within the Ascension Island EEZ, including the Harris Stewart Seamount, the Grattan Seamount, and the Young Seamount (Fig 1). Grattan and Young are sister peaks located *ca*. 80 km apart, on or adjacent to the mid-Atlantic ridge, and are jointly known as the “Southern Seamounts.” All the features studied lie 260 to 320 km offshore from Ascension Island, which itself lies more than 2,000 km from Africa—the nearest continental land mass (Fig 1). Detailed bathymetric mapping carried out during the study shows that Ascension’s seamounts can be divided into 2 groups along geographic lines. Grattan and Young are both shallow features rising to within 101 m and 77 m of the surface, respectively, with summits that intersect the deep chlorophyll maximum (the zone of peak primary production in tropical oceanic waters) and are subject to strong internal tides that drive semi-diurnal upwellings of cooler water from deeper strata (S1 Fig and Table A in S1 Text). The summit of the Harris Stewart seamount, in contrast, lies entirely within the mesopelagic “twilight zone,” consisting of a broad plateau at *ca*. 500 m depth punctuated by 2 distinctive domes that constitute the shallowest points at depths of 260 to 300 m (S1 Fig and Table A in S1 Text). These differences had a profound impact on their pelagic ecology.

### Are Ascension Island’s seamounts predator hotspots?

**Fig 2:**
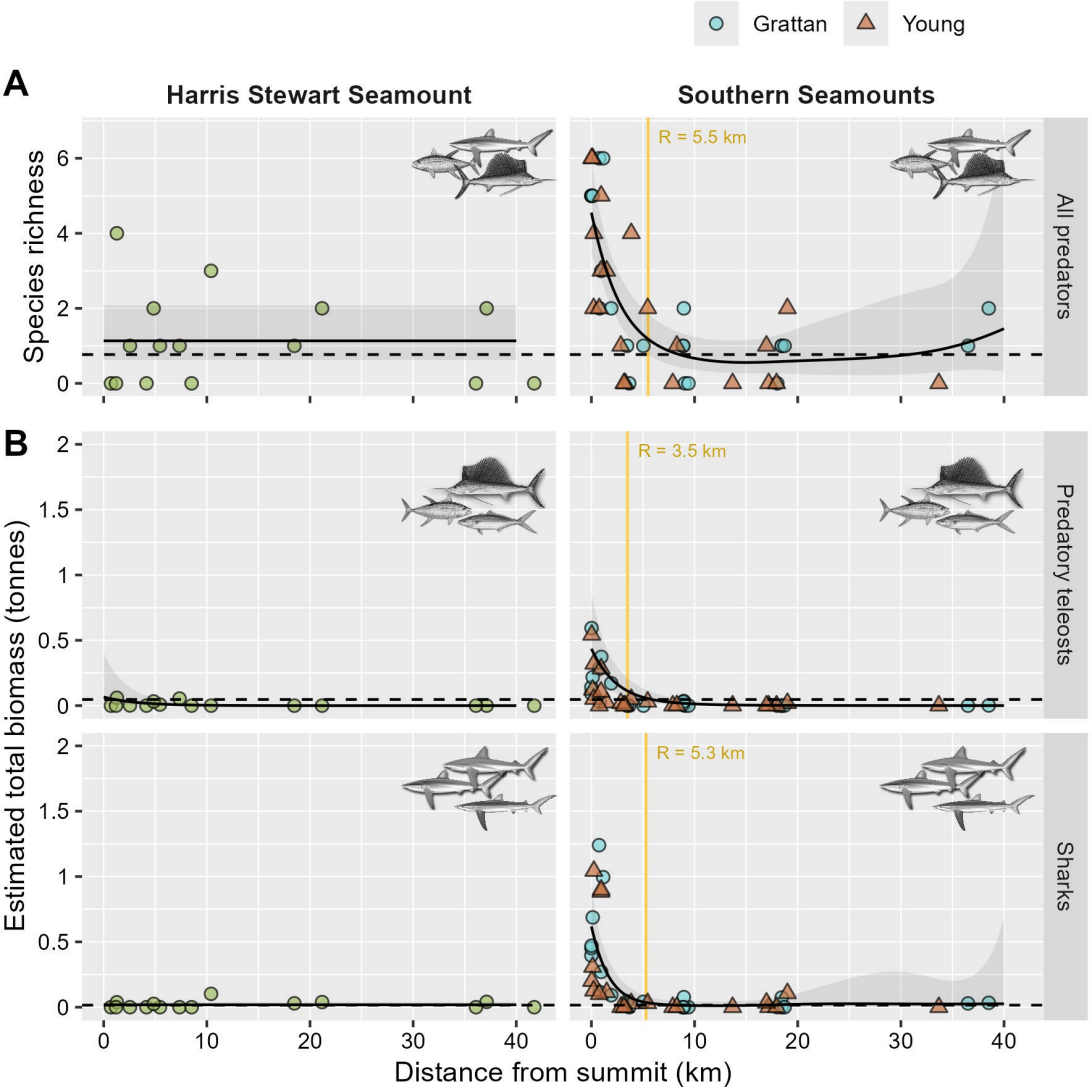
**Variation in (A) species richness and (B) estimated relative biomass of sharks and predatory teleosts (trophic level >4) observed in stereo BRUV surveys as a function of distance from Ascension Island’s outlying seamounts.** Estimated relative biomass is the maximum biomass of each taxa observed in a single video frame based on photogrammetric fork length measurements of individual animals converted using established length–weight relationships (see Methods). Solid trend lines and shaded envelopes are fitted smooths and associated 95% confidence intervals from negative binomial GAMs. Broken lines represent regional oceanic baselines from a reference set of 56 BRUVs surveys conducted >50 km from any seamount or island (see S1 Fig). Vertical gold lines represent the estimate radius of influence of the seamount where biomass or species richness began to singificantly increase and exceeded the regional baseline (see Methods). Data are plotted separately for the 2 Southern Seamounts but are modelled jointly based on AIC model selection which favoured a single global distance smoother for these adjacent shallow features in all cases. The data underlying this figure can be found in S2 Data. Illustrations Marc Dando (sharks) and Diane Rome Peebles (fish). AIC, Akaike’s information criterion; BRUV, baited remote underwater video.

Strong evidence of a predator-aggregating effect was apparent in baited remote underwater video (BRUV) and visual surveys conducted over the shallow Southern Seamounts, but not around the deeper Harris Stewart seamount (Fig 2). In total, 29 species of pelagic sharks, fish, and cetaceans were observed in BRUV transects (Table B in S1 Text). When considering only large-bodied predators (i.e., sharks and teleosts at trophic level >4), species richness was found to increase significantly within 5.4 km of the summits of the Southern Seamounts (GAM, *p* < 0.001; Table S3), rising from an average of 0.8 species per deployment (95% credible interval: 0.5 to 1.0) at distances >5.4 km, to 4.6 species per deployment (95% CI: 3.5 to 6.0) at the summit (Fig 2A). Predator biomass was also substantially elevated within 3.4 to 5.3 km of the Southern Seamounts (GAM, *p* < 0.001; Table S3), being 10.3 times higher for predatory teleosts (95% CI: 3.4 to 48.4) and 41.2 times higher for pelagic sharks (95% CI: 20.1 to 91.8) at the summits compared to mean biomass outside the seamount radius of influence (Fig 2B).

**Fig 3:**
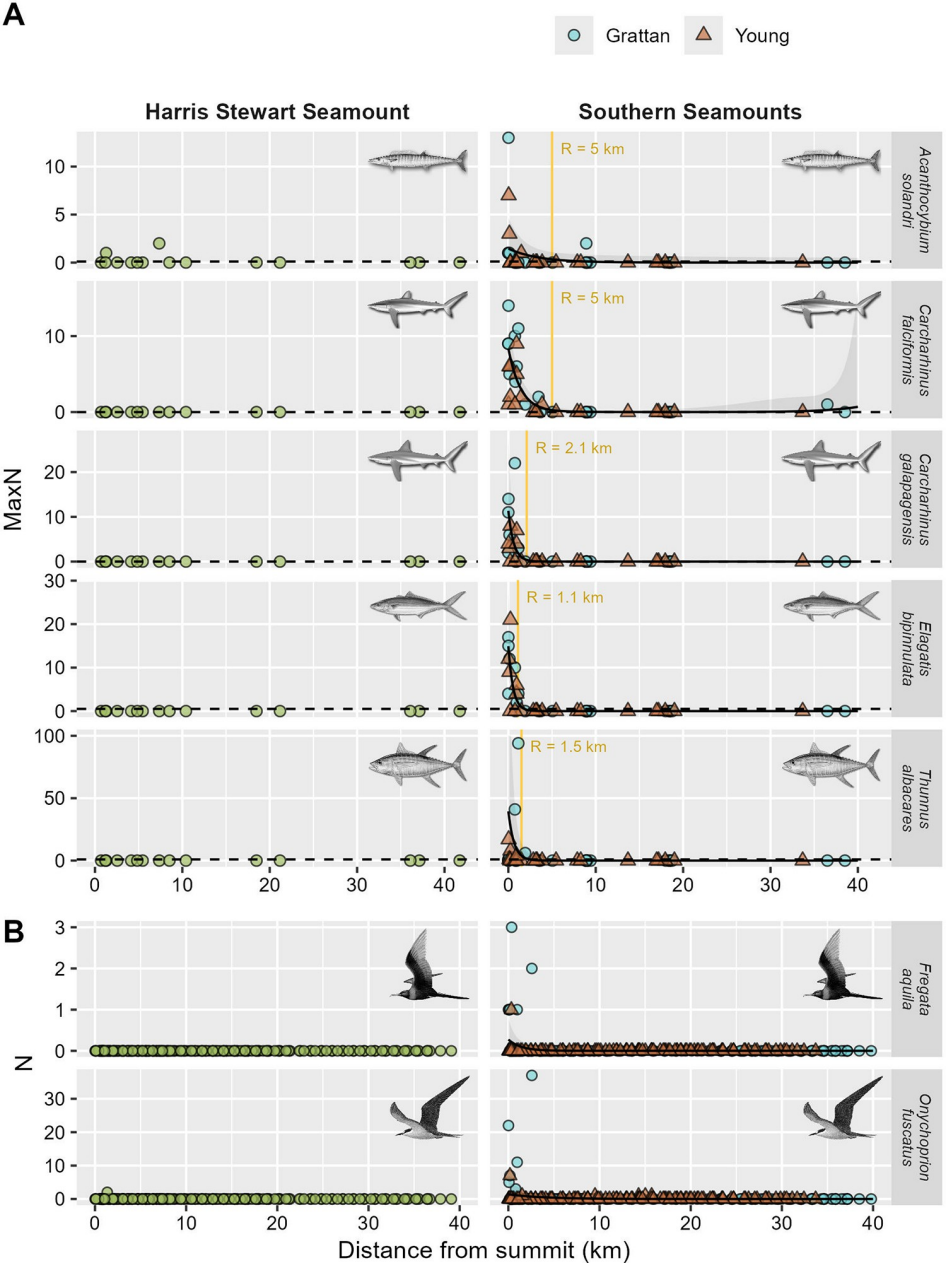
**Variation in relative abundance of predatory fish, sharks, and seabirds observed in (A) BRUVs surveys and (B) vessel-based visual transects as a function of distance from seamount summits.** Relative abundances are expressed as the maximum number of individuals observed in a single video frame for each BRUV deployment (MaxN) and as the number of individuals observed in a 300 m belt transect per 5-min sampling window for visual transects. Solid trend lines and shaded envelopes are fitted smooths and associated 95% confidence intervals from negative binomial GAM(M)s. Only species exhibiting significant seamount associations are shown (see S3 and S4 Figs for all species). Broken lines and gold bars in (A) represent the regional oceanic baseline and approximate radius of influence of the seamount for each species as per Fig 2. The data underlying this figure can be found in S2 Data (A) and S3 Data (B). Illustrations Marc Dando (sharks), Diane Rome Peebles (fish), and Peter Harrison (seabirds). BRUV, baited remote underwater video.

However, shallow seamounts did not aggregate all pelagic predator species equally. Of the 8 most frequently observed species in BRUV surveys (those present in ≥6 deployments), 5 were found to be significantly more abundant within 1 to 5 km of the Southern Seamounts, including silky sharks (*Carcharhinus falciformis*), Galapagos sharks (*Carcharhinus galapagensis*), yellowfin tuna (*Thunnus albacares*), rainbow runner (*Elagatis bipinnulata*), and wahoo (*Acanthocybium solandri*) (GAM, all *p* ≤ 0.037; Fig 3A and Table C in S1 Text). Abundances of blue shark (*Prionace glauca*), mahi mahi (*Coryphaena* sp.), and sailfish (*Istiophorus albicans*) did not vary significantly with distance from the summits of these features over the scales sampled by our radial transects (GAM, all *p* > 0.1; Table C in S1 Text and S3 Fig). However, when considering all deployments made within 20 km of the Southern Seamounts (*n* = 41), the probability of occurrence of sailfish was an order of magnitude higher than regional oceanic baselines, which may indicate a looser association (seamount probability = 0.20; oceanic probability = 0.018; Z test: $\chi_{1}^{2}$ = 6.9, *p* = 0.009) (S3 Fig and Table B in S1 Text). Similarly, mixed results were found in vessel-based visual surveys. Of the 7 seabird species observed in visual transects (Table D in S1 Text), only 2—the Ascension frigatebird (*Fregata aquila*) and the sooty tern (*Onychoprion fuscatus*)—showed evidence of local enhancement around seamounts, with elevated abundances detected within ca. 2 km of the summit (GAMM, all *p* ≤ 0.035; Fig 3B and Table E in S1 Text). Other commonly observed species such as storm petrels (*Oceanodroma* sp.) and shearwaters (*Procellariidae* sp.) exhibited no significant associations with seamounts (GAMM, all *p* > 0.7; S4 Fig and Table E in S1 Text). No cetacean species were observed in visual transects and only a single individual was detected in BRUV surveys suggesting that, unlike some seamounts [10], our study features are not important aggregation areas for these species (at least during the sampling period).

No evidence of elevated predator species richness, total predator biomass, or predator abundance were found in comparable BRUV surveys and visual transects conducted over the deeper Harris Stewart seamount (GAM, all *p* > 0.11; Figs 2 and S6 and Table G in S1 Text), although only a single species, blue shark (*Prionace glauca*), was observed sufficiently frequently to analyse abundance trends. A weak positive trend in biomass of predatory teleosts was detected in BRUV transects conducted over this feature (GAM, *p* = 0.006); however, the predicted biomass at the summit was below the regional oceanic baseline (Fig 2).

### Can seamount predator hotspots be explained by an “oasis effect”?

#### Mid-trophic level biomass

**Fig 4:**
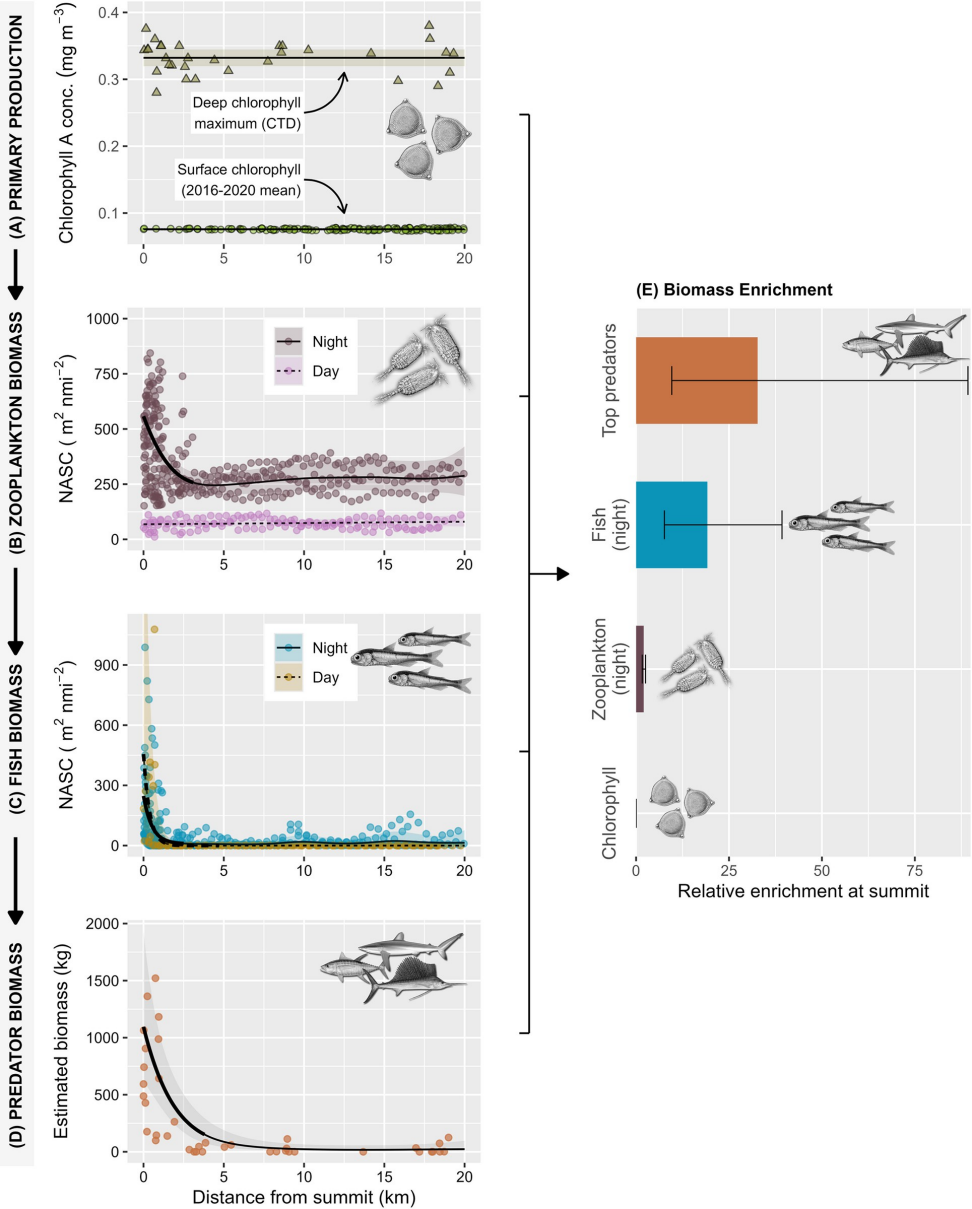
The “oasis effect” of Ascension Island’s Southern Seamounts across multiple taxa and trophic levels. Panels (A–D) show contemporaneous seamount–distance relationships for: **(A)** The deep chlorophyll maximum recorded in CTD profiles, along with pluriannual mean sea surface chlorophyll concentration derived from satellite data; **(B, C)** hydroacoustic NASC of **(B)** zooplankton and **(C)** fish integrated over the top 300 m of the water column for each 500 m horizontal sampling unit; and **(D)** total biomass of large pelagic fish (trophic level >4) and sharks in mid-water baited remote underwater video surveys. Trend lines and associated 95% confidence intervals (shaded envelopes) in A–D are thin plate regression splines fit using GAMs/GAMMs with data-appropriate error distributions and autocorrelation structures (see Methods). For consistency, plotted trends are global smooths fit to pooled data from both Southern Seamounts as model comparisons favoured this approach over seamount-specific smooths in all cases except for pelagic fish NASC (see S5 Fig). Emboldened sections of each smooth show regions of significant change based on analysis of model derivatives (see Methods) and define the seamount radius of influence (*R*). Panel **(E)** shows the estimated relative enrichment of each taxon at seamount summits (mean at distance = 0) compared to pelagic baselines (mean at distance > R), along with 95% credible intervals. The data underlying this figure can be found in S2 Data (D), S4 Data (B, C), and S5 Data (A). Illustrations: (A–C) Creative Commons via Wikimedia, (D) Marc Dando (sharks), and Diane Rome Peebles (fish). CTD, conductivity-temperature-depth; NASC, nautical area scattering coefficient.

**Fig 5:**
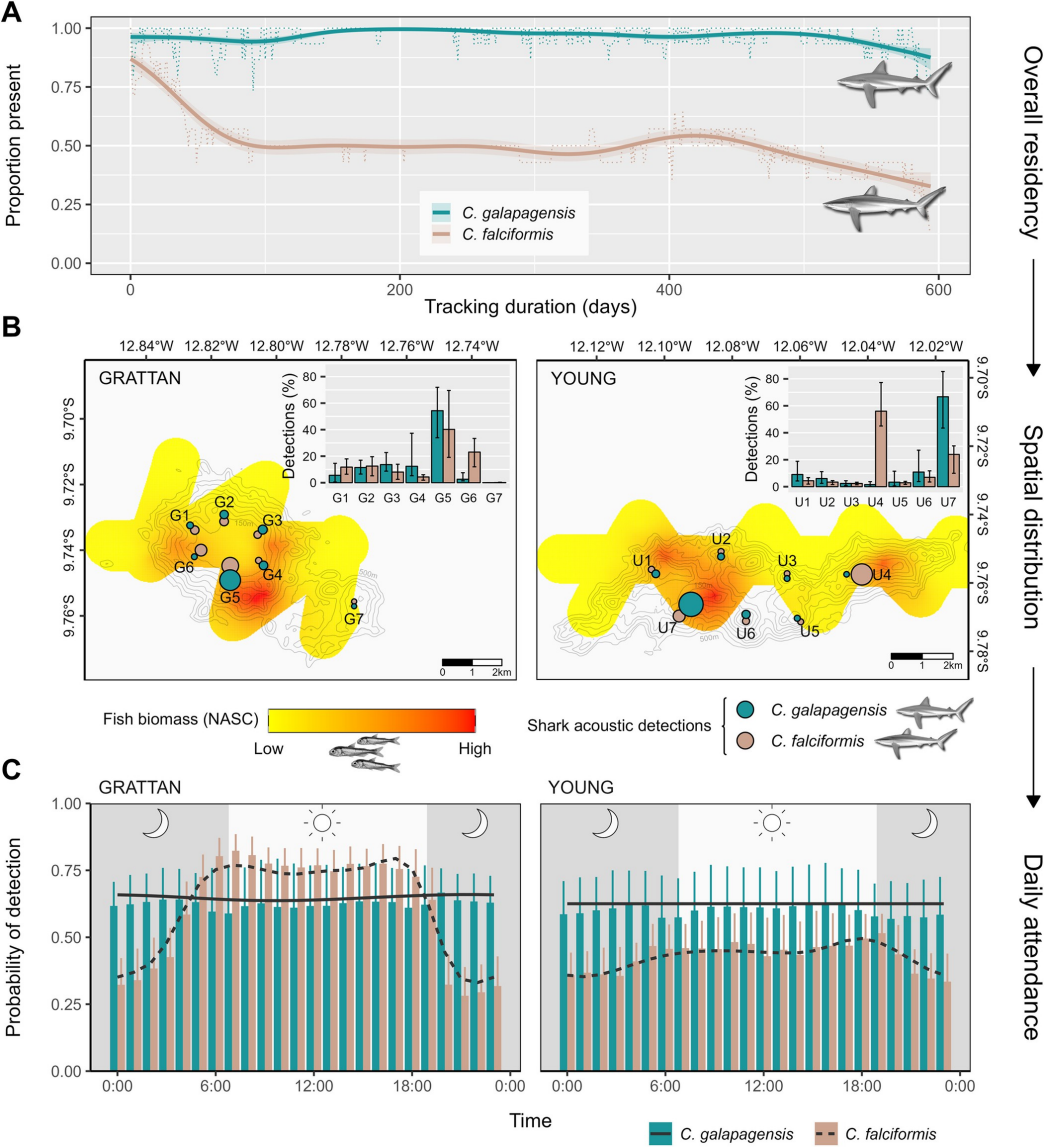
Residency and space use of acoustically tagged Galapagos and silky sharks on Ascension Island’s Southern Seamounts. (A) Attrition curves showing how the proportion of tagged individuals detected at least once on seamount summit receiver arrays varied over the study period (June 2017–January 2019). Broken lines show raw proportions and solid lines show fitted trends from binomial GAMs with an AR(1) autocorrelation structure. (B) Spatial distribution of tagged sharks during periods of residency on seamounts. Map symbols show acoustic receiver locations (offset by species for clarity) and are scaled according to the mean proportion of detections recorded at each site across the study period. Mean proportions and bootstrapped 95% confidence intervals are also presented in inset barplots. Shark distributions are overlaid on heatmaps of total fish biomass derived from ordinary kriging of nocturnal hydroacoustic NASCs collected in June 2017 (see S4 Fig for details). (C) Diel variation in detection probability of tagged sharks during periods of residency on seamounts. Detection probabilities are expressed as the proportion of hourly bins in which an animal was detected at least once on summit receiver arrays, calculated across all days when an individual was present. Trend lines are fitted smooths from quasibinomial GAMMs with a random effect of shark ID. The data underlying this figure can be found in S6 Data. Illustrations: Marc Dando (sharks); fish from Wikimedia under Creative Commons License. NASC, nautical area scattering coefficient.

Hydroacoustic transects carried out in parallel with predator surveys provided some support for the “oasis hypothesis,” with evidence of significantly enhanced mid-trophic faunal biomass around the summits of the Southern Seamounts (Fig 4B and 4C). This enrichment was particularly apparent at night when vertically migrating species ascended into shallower waters. Fitted GAMMs indicate that nocturnal biomass of zooplankton and pelagic fish began to increase significantly within 3.2 km and 2.4 km of the Southern Seamounts (all *p* < 0.001; Table F in S1 Text), respectively, and was on average 2 times higher for plankton (95% CI: 1.6 to 2.5) and 19.2 times higher for fish (95% CI: 7.6 to 39.3) at the summits when compared to pelagic baselines (Fig 4). Zooplankton trends were comparable for the 2 Southern Seamounts; however, increases in nocturnal fish biomass were more pronounced on Grattan (28-fold) than Young (11-fold) (Table F in S1 Text and S5 Fig). Hydroacoustic surveys also demonstrate that faunal biomass was not evenly distributed around seamount summits but instead tended to be concentrated in localised hotspots, including the southern flank of Grattan and on several subpeaks of Young (S6 Fig). These hotspots broadly corresponded with activity centres of acoustically tagged Galapagos and silky sharks tracked across the same period, suggesting some trophic interaction or common aggregating mechanism (Fig 5B). Elevated nocturnal biomass of fish and zooplankton was also evident over the Harris Stewart seamount, although the increase was only significant for fish and was highly localised over the summit domes (S5 Fig and Table F in S1 Text).

In addition to driving overall pelagic biomass enrichment, seamounts also influenced the vertical distribution of prey biomass in the water column. As is typical in the tropical pelagic zone, at night, fish and zooplankton were generally concentrated in a well-defined scattering layer that corresponded closely with the deep chlorophyll maximum at approximately 100 m depth (S7 Fig). However, this scattering layer weakened within *ca*. 5 km of seamount summits and nocturnal biomass enhancement was instead driven by deeper accumulations of plankton and fish associated with the upper slopes at depths of 120 to 200 m on the Southern Seamounts and 260 to 280 m over Harris Stewart (S7 Fig). During daylight hours, many vertically migrating taxa descend into deeper mesopelagic waters and correspondingly we found no seamount-enhancement effect for zooplankton in diurnal hydroacoustic surveys (GAMM, *p* = 0.47; Fig 4 and Table F in S1 Text). The vertical distribution of fish also shifted deeper during the day; however, significant accumulations of fish were still detected close to the summits of the Southern Seamounts (GAMM, *p* < 0.002; Fig 4 and Table F in S1 Text) where they tended to be concentrated in dense schools associated with the seabed at depths of 250 to 300 m (S7 Fig). These results suggest that low and mid-trophic biomass enrichment over seamounts was primarily driven by vertically migrating mesopelagic species. Consistent with this, we found no evidence of elevated biomass of epipelagic forage fish over seamount summits in shallow diurnal BRUV surveys (GAM, *p* = 0.27; Table C in S1 Text and S8 Fig) while the relative abundance of flying fish (*Excoetidae sp*.)—an important epipelagic prey taxa—observed in visual transects declined marginally over the Southern Seamounts (GAMM, *p* = 0.045; Table E in S1 Text and S8 Fig).

#### Primary productivity

Despite supporting significantly elevated faunal biomass, we found no evidence of a chlorophyll enrichment effect around any of Ascension’s seamounts. The concentration and depth of the deep chlorophyll maximum did not vary significantly with distance to seamount summits in CTD transects carried out concurrently with biodiversity surveys (GAM, all *p* > 0.11; Fig 4 and Table G in S1 Text). Similarly, mean sea surface chlorophyll concentrations extracted from remote sensing data over a 5-year period spanning our study (2016 to 2020) were also not locally elevated over seamount summits (GAM, *p* > 0.15; Figs 4 and S9 and Table G in S1 Text). Instead, local climatologies were dominated by a strong latitudinal gradient, with mean primary productivity being highest to the north of all study seamounts (S9 Fig). When this background mesoscale variability was removed through calculation of a daily “chlorophyll enrichment index” ([24]; see Methods), an increasing trend emerged, particularly over the Young Seamount (Table G in S1 Text and S9 Fig). However, the estimated enrichment index at the summit was <0.1% (S9 Fig). Thus, while shallow seamounts had marginally higher surface chlorophyll concentrations than would be expected given their geographic location, mean absolute primary productivity was comparable to or lower than surrounding oceanic waters and cannot satisfactorily explain the substantial increases in biomass observed at higher trophic levels.

Indeed, a notable finding from our integrated surveys is that pelagic biomass enrichment around Ascension’s shallow seamounts appeared to increase with trophic level, ranging from a 2-fold increase for zooplankton to a 35-fold increase for top predators (Fig 4E). Such “top-heavy” systems can arise through various mechanisms, including the movement of consumers across ecosystem boundaries [25], warranting a more detailed investigation into the interactions of individual top predators with seamounts.

### Are seamount predator aggregations comprised of resident populations or transient visitors?

To investigate how the dominant pelagic predators observed in our surveys interact with seamounts, a combination of passive acoustic and satellite telemetry was used to study the movements of individual Galapagos sharks (*n* = 15), silky sharks (*n* = 14), yellowfin tuna (*n* = 9), and bigeye tuna (*Thunnus obesus*, *n = 9*) captured from summit aggregations (Tables H and I in S1 Text). Although the latter were not observed sufficiently frequently in shallow diurnal BRUV surveys to model abundance trends (probably because they spend the day foraging in mesopelagic waters [26]), this deeper-schooling species is known to associate with seamounts [10,22] and was regularly caught during dawn/nocturnal fishing around our study features.

#### Sharks

Galapagos and silky sharks tagged with acoustic transmitters over the Southern Seamounts consisted predominantly of juveniles and subadults (mean ± SD fork length, Galapagos: 127 ± 23.5 cm, range = 105 to 196 cm; silky: 125 ± 14.3 cm, range = 102 to 152 cm; Table S8), which is consistent with size ranges estimated from BRUV surveys (silky sharks: 138 ± 22.5 cm; Galapagos sharks: 158 ± 21.0 cm; Table S8). Detections recorded by summit hydrophone arrays reveal that Galapagos sharks were almost continuously resident over the 595-day tracking period and had significantly higher mean residency indices (RI = proportion of tracking days with ≥1 detection) than silky sharks, which were more likely to disperse away during early phases of the study (Galapagos: mean RI = 0.97; Silky shark: mean RI = 0.68; quasibinomial GLM: *F*_1,28_ = 23.7, *p* < 0.001) (Fig 5A). The proportion of tagged silky sharks remaining on the Southern Seamounts declined rapidly over the first 90 days, but then stabilised at *ca*. 50% for the remainder of the study (Fig 5A). Partitional clustering around medoids identified 2 distinct groups of silky sharks which corresponded with this biphasic curve: those with RI ≤ 0.34 (“transients”; *n* = 7) and those with RI ≥ 0.76 (“residents”; *n* = 7) (Fig 3). Neither fork length nor sex influenced the observed RIs (quasibinomial GLM: all *F* < 1.1, *p* > 0.31). Galapagos and silky sharks also behaved differently during periods of residency on seamounts. While the former exhibited high and relatively constant mean detection probabilities across the diel cycle on both Southern seamounts (GAM, Grattan: *F* = 0.53, *p* = 0.042: Young: *F* = 0.0, *p* = 0.39), silky sharks displayed a strong circadian rhythm, with detections declining shortly after dusk and returning before dawn (GAM, all F > 16.2, *p* < 0.001; Fig 5C).

**Fig 6:**
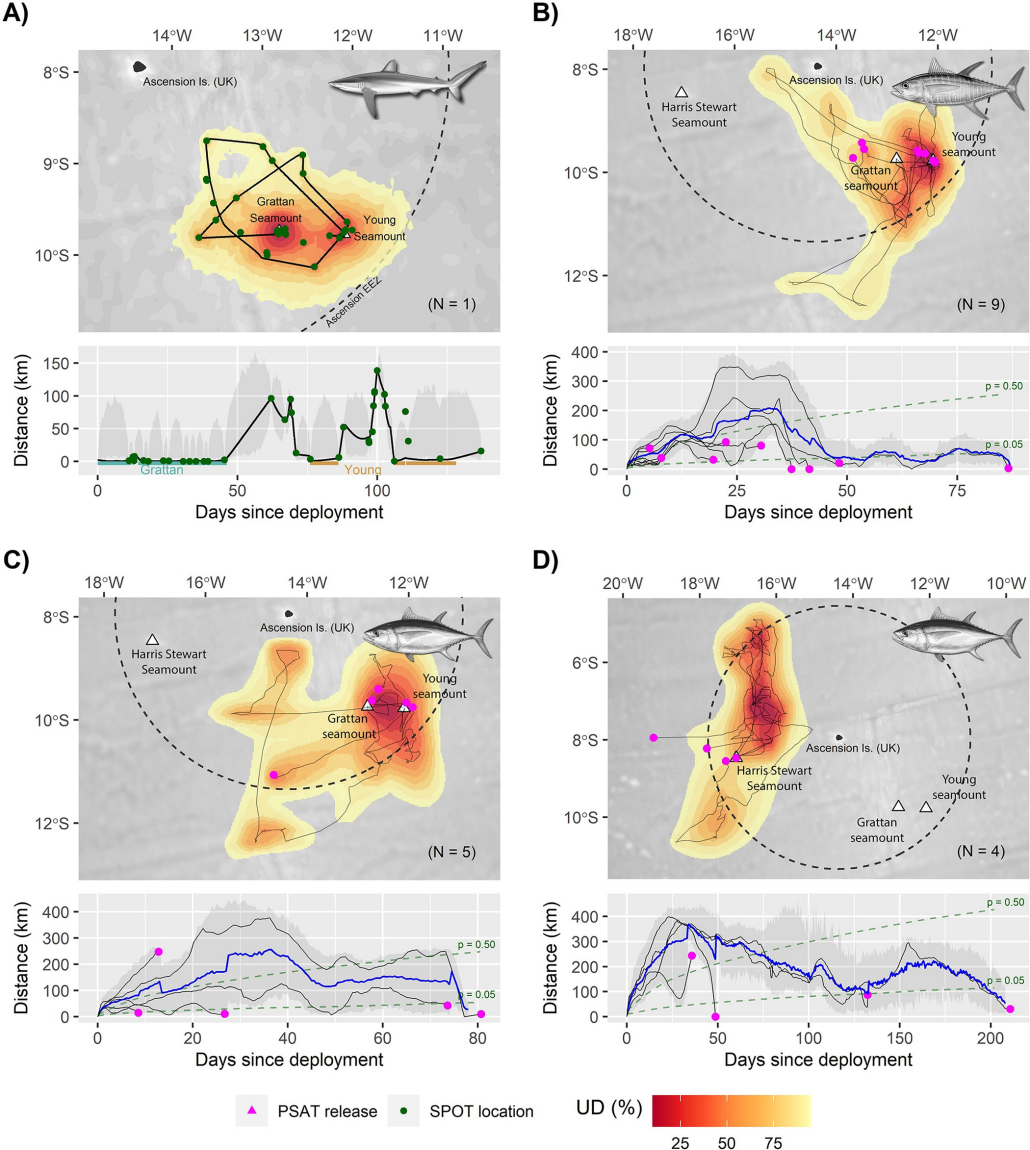
Movements and site fidelity of satellite-tagged sharks and tuna captured at the Grattan, Young and Harris Stewart seamounts. (A) Movements of a single silky shark (*C*. *falciformis*) tagged with a fin-mounted Argos-GPS SPOT transmitter on the Grattan Seamount in June 2017. (B–D) Movements of (B) 9 yellowfin tuna (*T*. *albacares*) and (C, D) 9 bigeye tuna (*T*. *obesus*) fitted with PSATs at the Southern Seamounts (B, C) and Harris Stewart Seamount (D) between May 2017 and February 2018. In each case, upper panels show the posterior mean tracks of tagged individuals along with the overall “utilisation distribution” (UD), of the population derived from tag-specific SSMs (see Methods). UDs are presented as volume contours enclosing a given proportion of the joint SSM posterior probability density and were calculated using a weighted average that gives greater influence to individuals with longer deployment periods. Lower panels show how distance from the tagging seamount(s) varied with time for each posterior mean track, along with the population mean (blue line; tuna only) and associated 95% confidence interval (shaded regions) derived from SSM posterior simulations. Broken lines in lower panels (B–D) are isopleths describing the probability (*p*) that a hypothetical individual would have been located within a given distance of their tagging seamount on each relative tracking day if they dispersed randomly. Probabilities were estimated using correlated random walk simulations (*n* = 10,000) and constitute a test of the null hypothesis that tagged animals showed no fidelity to seamounts. The data underlying this figure can be found in S6 Data. Illustrations: Marc Dando (sharks) and Diane Rome Peebles (tuna). Bathymetry basemaps were sourced from GEBCO. PSAT, pop-up satellite archival tag; SSM, state-space model.

Unfortunately, locations from sharks double-tagged with fin-mounted GPS tags (*n* = 9 per species) were generally too sparse to reconstruct their movements during periods of absence from seamount summits. However, regular transmissions over a 137-day period by a single silky shark were potentially revealing. Following an initial period of residency on the Grattan seamount lasting 46 days, this individual ranged extensively over the south-eastern quadrant of the Ascension EEZ, making 2 return visits to the Young seamount after absences of 20 to 30 days during which it travelled up to 140 km from the seamount summits (Fig 6A). Similar movements between Grattan and Young were also apparent in detections of acoustically tagged animals. While fidelity to the seamount on which tagging originally occurred was generally high, 5 (36%) silky sharks and 2 (13%) Galapagos sharks moved between the Southern Seamounts at least once during the study (S10 Fig).

#### Tuna

Yellowfin tuna (85 to 134 cm fork length, *n* = 9) and bigeye tuna (97 to 165 cm, *n* = 5) tagged with pop-up satellite archival tags (PSATs) on the Southern Seamounts exhibited residency behaviours that superficially resembled those of silky sharks (Fig 6B and 6C). Both species demonstrated some fidelity to the Southern Seamounts over their respective tracking periods (bigeye: 9 to 77 days; yellowfin: 5 to 87 days; Table I in S1 Text): utilisation distributions were strongly centred on the seamount peaks and all but one individual with tracking durations >25 days (*n* = 9) were located significantly closer to seamounts at the point of tag release than expected under random dispersal (range = 0 to 43 km, all *p* < 0.05) (Fig 6). However, most probable tracks also suggest that both species engaged in extended migrations away from the Southern Seamounts, travelling distances of up to 400 km. These broader scale movements must be interpreted with caution due to the low spatial resolution of PSAT geolocations. Nevertheless, 3 yellowfin tuna tagged on the Grattan Seamount were confidently located 72 to 93 km to the northwest when their tags released 5 to 31 days later, while 1 bigeye tuna had travelled *ca*. 250 km to the southwest over a 13-day period prior to shedding its tag (Fig 6B).

Bigeye tuna (115 to 120 cm, *n* = 4) tagged on the deeper Harris Stewart seamount also appear to have embarked on long post-tagging migrations (Fig 6D). Unlike conspecifics tagged on the Southern Seamounts, the utilisation distribution of these individuals was not centred over the peak, instead implying extensive use of an area to the northwest of Ascension Island that was previously the focus of a commercial longline fishery targeting this species [27]. However, 2 individuals were still located significantly closer to the Harris Stewart seamount than expected under random dispersal when their tags released 49 and 211 days later (1 and 30 km, respectively; *p* < 0.01), indicating that they either returned to it or remained more closely associated than geolocator tracks imply (Fig 6D).

## Discussion

Our results provide further evidence of the importance of shallow seamounts for pelagic top predators in one of the world’s largest MPAs and offer fundamental insights into their functional roles as prey oases and activity hubs for these species. Compared to surrounding oceanic waters, Ascension Island’s shallow Southern Seamounts supported 5 times higher diversity and 30 times higher biomass of sharks and large predatory fishes, with significantly elevated abundance of several threatened/near-threatened (e.g., Galapagos and silky sharks; IUCN 2024) and commercially exploited species (e.g., yellowfin and bigeye tuna). Individuals of these species were also found to be highly resident over periods of months (tunas) to years (sharks) which may render them both vulnerable to local exploitation [28] and potential beneficiaries of area-based protection measures [29]. These findings contributed to evidence that informed the designation of the Ascension Island MPA in 2019. However, our results also corroborate results from previous studies showing that not all seamounts aggregate pelagic predators in the same way, with summit depth being a key factor [10]. While the deeper Harris Stewart Seamount revealed evidence of localised biomass hotspots at lower trophic levels, these were confined to cold, sub-thermocline waters and the aggregations of epipelagic apex predators that dominated the Southern Seamounts were conspicuously absent. Intermediate depth seamounts such as Harris Stewart have been shown to attract mesopelagic predators (e.g., bigeye tuna) [22] that would have low detectability in our shallow BRUV surveys, meaning we cannot rule out a predator aggregating effect deeper in the water column. Nevertheless, summit depth was evidently fundamental in structuring seamount predator assemblages.

The spatially predictable abundance and diversity of top predators found around many shallow seamounts, along with the high residency exhibited by some individuals, makes them natural focal points for area-based protection in the open ocean [6]. In the case of Ascension Island, economic considerations ultimately led to a decision to close the entire EEZ to commercial fishing in 2019 [30]. However, a key conservation question raised by studies such as this is: How large should seamount MPAs be to adequately protect associated predator hotspots? In the current study, evidence of a significant predator enrichment effect was detectable up to 6 km from the summits of shallow seamounts. This is somewhat lower than the 10 to 40 km reported by previous studies [2,10], which may be partly explained by the different sampling methods used. For example, longline catch data used by previous meta-analyses [2] are generally aggregated by sets that can be tens of kilometres long, limiting ability to model fine scale trends. Conversely, the fisheries-independent methods used in the current study permit significantly lower sampling effort than can be achieved using commercial fisheries catches, which may have limited our power to detect weak seamount effects at greater distances. Indeed, several aspects of our results suggest that our estimates of seamount radius of influence are conservative. For example, abundances of sailfish peaked at intermediate distances (ca. 5 to 10 km) from the Southern Seamounts and were substantially higher than in oceanic reference surveys; individual silky sharks, yellowfin tuna, and bigeye tuna ranged hundreds of kilometres from seamount summits while remaining ostensibly “site-attached”; and acoustically tagged Galapagos and silky sharks travelled the *ca*. 80 km between the Southern Seamounts sufficiently frequently for them to be considered as a single “system” for the purposes of shark conservation. Juvenile silky sharks observed in diurnal BRUV surveys also moved off seamount summits at night, suggesting “halos” of enhanced predator abundance that expand and contract across the diel cycle. Collectively, these findings provide strong justification for the establishment of larger pelagic MPAs centred on seamounts—and particularly on seamount chains or constellations—that protect functional connections between neighbouring features and adjacent deep-water ecosystems.

Aside from their conservation significance, large predator aggregations over seamounts also pose fundamental questions about the biophysical mechanisms that sustain them. Overall, our results provide strong support for the “oasis” hypothesis. The shallow Southern Seamounts, in particular, revealed evidence of systematic enrichment of faunal biomass across the food web, from plankton to apex predators, suggesting that predictable concentrations of prey may be a major draw for pelagic megafauna. However, the drivers of this trophic enrichment are less clear. Topographically induced upwelling appears to be a common phenomenon around seamounts and other bathymetric features [31,32] and can transport limiting nutrients into the euphotic zone [14,33]; however, evidence that this translates into locally enhanced primary productivity is mixed [15,16]. Indeed, despite evidence of tidal upwelling over the summits of the Southern Seamounts, we found no or very weak evidence of enhanced chlorophyll A concentrations, either at the time of our surveys or in multi-year satellite imagery composites. One explanation is that, in the absence of some form of retaining circulation (e.g., Taylor caps), advected nutrients are rapidly exported downstream by currents before they can promote phytoplankton growth [13,16]. Thus, while seamounts are clearly capable of stimulating primary production [15], our results suggest that this effect is not universal and is not necessary for an “oasis effect” at higher trophic levels.

Instead, our results are more consistent with the hypothesis that seamount predator assemblages are sustained by trophic subsidies from exogenous sources [16]. Indeed, an interesting observation from our integrated surveys is that pelagic biomass enrichment over the summits of shallow seamounts appears to increase with trophic level, ranging from a 2-fold increase for zooplankton to a 41-fold increase for pelagic sharks. Although absolute biomass estimates from our different survey methods are not directly comparable, these relative increases suggest that seamounts support a greater abundance of large predators than would be expected based on low- and mid-trophic pelagic biomass enrichment alone. Similar “inverted,” or predator dominated, trophic pyramids have been previously reported in other pristine (“unfished”) marine systems associated with shallow topographic features [25,34] and are hypothesised to arise through several mechanisms, including trophic subsidies from movements of both consumers and nutrients across community boundaries [25]. The latter is equivalent to “trophic focussing” [16], which posits that planktonic prey swept over seamounts becomes concentrated on the summits through DVM trapping and capture by benthic consumers. Paradoxically, the principle mechanisms thought to drive trophic focussing do not predict higher zooplankton biomass on seamounts; rather, planktonic biomass may be lower over the summit as benthically trapped layers are depleted by consumers during the day and must be replenished at night [16]. A weakening of the nocturnal scattering layer was apparent over the summits of our study features, but this was more than compensated by zooplankton accumulations at deeper depths. Additional sampling of these biomass plumes is needed to determine whether they are comprised of cosmopolitan pelagic species or resident seamount “specialists” [16,35]. Since none of our estimates of trophic biomass enrichment included benthos, further research into seamount food webs and energy flows is needed to assess the extent to which trophic focussing and bentho-pelagic coupling contribute to high observed pelagic predator densities.

A second, non-mutually exclusive source of trophic subsidies is that, rather than concentrating prey, seamounts concentrate predators that forage elsewhere and thus integrate resources gathered over a wider area [25]. Several aspects of our predator-tracking results are consistent with this “hub hypothesis.” In the longer term, both silky sharks and tunas revealed evidence of extended periods of absence from seamounts during which they ranged extensively throughout the Ascension EEZ before subsequently returning. Over diel timescales, patterns of acoustic detections also suggest that “resident” silky sharks frequently moved off seamounts at night when epipelagic prey is abundant before returning at dawn. Similar central-place foraging behaviour has been reported in hammerhead sharks (*Sphyrna* spp.) on seamounts [20,36,37] and in silky sharks associated with various topographic features [21,38], and may help to explain the predator-dominated biomass pyramids commonly found around unfished topographic features [25,34], much as seabird colonies create artificially high densities of top predators that cannot be sustained by locally available resources alone. The benefits of central place foraging for sharks is unclear but may include sustained feeding opportunities, “cleaning services” solicited from feature-attached demersal fish [19], opportunities for social information exchange [39], or energy conservation from associating with hydrodynamic features over seamounts [40]. Interestingly, Galapagos sharks displayed no consistent diel cycling and were highly resident and site-attached throughout the study, suggesting that these species occupy different ecological niches that facilitate coexistence on seamounts (see [41]). However, during periods of occupancy on seamounts, both silky and Galapagos sharks were spatially aggregated in localised hotspots that were consistent with areas of high mid-trophic fish biomass, implying some common aggregating mechanism. Similar aggregations have often been reported in sharks associated with topographic features (so-called “hotspots within hotspots” [42]) and more detailed investigations into the local hydrodynamic processes that create them would likely offer valuable insights into the overall mechanisms that make shallow seamounts critical habitats for many pelagic top predators.

Finally, it is important to note that while overall predator biomass and diversity was significantly elevated over Ascension’s shallow seamounts, not all predator species interacted with these features in the same way. Similar findings have been reported elsewhere [2,10] and may be partly explained by differences in predator foraging ecology. For example, among the more commonly observed species in our surveys, mahi mahi and some oceanic seabird species (shearwaters and storm petrels) did not exhibit significant association with seamounts, while seabirds in general were present in low numbers. All these species are known to be surface-orientated foragers that rely heavily on epipelagic prey [26,43] that were not found to be significantly enriched over Ascension’s shallow seamounts. Instead, pelagic biomass enrichment was primarily driven by vertically migrating mesopelagic species, suggesting that predator diet and depth use is likely be important in determining seamount use. It is also notable that the 2 seabird species that were locally more abundance over shallow seamounts (Ascension frigatebird and sooty tern) are known to be near-obligate commensals of surface-schooling predatory fish (e.g., tuna), suggesting that they may have been attracted by “facilitated foraging” with seamount-associated aggregations of these species [44]. Such non-trophic interactions among predator species may be an additional and relatively unstudied mechanism through which seamounts aggregate pelagic top predators.

In the UN Decade of Ocean Science, the need for evidence-based marine management has never been clearer. Recognising the unprecedented threat faced by marine ecosystems, many conservationists and world leaders have called for ambitious targets of protecting 30% of our oceans by 2030 [45], posing challenging questions about which areas to prioritise and the effectiveness and enforceability of large, oceanic MPAs. As spatially predictable hotpots of abundance and diversity for site-attached pelagic predators, seamounts will continue to provide credible focal points both for the creation of “blue water” marine reserves and the delivery of targeted surveillance activities. Here, we have shown how the integrated application of a set of non-destructive research tools can be used to rapidly build a scientific case for seamount MPAs as well as contributing to our knowledge of how these remarkable habitats influence the oceans that surround them.

## Methods

### Ethics statement

All work was carried out under an Environmental Research Permit and Protected Wildlife Research License issued by the Ascension Island Government and approved by the Director of Conservation and Fisheries and the Ascension Island Administrator (reference ERP-2017-08). This also constitutes the relevant institutional ethical approval for crown employees of the Ascension Island Government who led the work.

### Mid-water stereo baited remote underwater video (BRUV) surveys

Midwater BRUV surveys were conducted using drifting camera assemblies that have been described in detail elsewhere [3,46]. Each rig consisted of a stereo pair of high-definition cameras (GoPro HERO3) trained on a bait canister filled with 1 kg of macerated tuna sourced as waste from a local fish processing facility. Rigs were deployed in sets of 5, suspended at a depth of 10 m and spaced 200 m apart along short, floated longlines. All deployments took place during the daytime (07:00 to 18:00) with a soak time of 2 h, during which each rig gathered continuous video footage. Video data were analysed using EventMeasure (https://www.seagis.com.au/event.html) to identify, count, and measure individuals crossing the field of view. Camera rigs were photogrammetrically calibrated to estimate the lengths of individuals, which were in turn converted to mass estimates using published weight-length relationships (see [46] for details). To avoid double counting (within and between rigs), the relative abundance and biomass of each species encountered was calculated as the maximum number (MaxN) or maximum combined mass (of individuals observed in a single frame when pooled across all rigs in a set). These metrics therefore represent the minimum abundance and biomass of each taxa present in the survey area. For each set, we also calculated the species richness as the total number of species for which at least one individual was observed. The majority of BRUV surveys were carried out during the principal seamount expedition in May to June 2017. However, 9 of 59 (15%) deployments (3 per seamount)—primarily over summit areas—occurred during scoping and follow-up visits between 20th January to 20th February 2017 and 2018. We therefore included “season” as a factor in all subsequent models. These visits formed part of larger research cruises during which more extensive BRUV sampling using identical methods was undertaken in oceanic waters (>50 km from any island or seamount) throughout the Ascension EEZ (*n* = 56 surveys; S2 Fig). We did not include oceanic samples in models of seamount distance effects due to the very large spatial scales involved (up to 560 km from summits). However, means and CIs across all oceanic deployments were used to define regional baselines for estimating seamount radius of influence (see Modelling).

### Vessel-based visual transects

Vessel-based counts of surface-orientated marine vertebrates, including seabirds and flying fish, were carried out as described in [43]. Surveys were performed by a single observer stationed on the bow of a 22 m vessel (*MV Extractor*) travelling at a constant speed (approximately 8 knots) and heading and involved counting all individuals observed in flight or at the surface in a belt transect approximately 300 m wide (i.e., visible to the naked eye with identifications confirmed by binocular if necessary). Individual transects were approximately 1 h in duration (range = 0.4 to 1.5) and covered an average distance of 14.6 km. To enable finer-scale analysis of seamount effects, within transects, counts of each species were pooled in 5-min observation intervals which formed the response variables for subsequent statistical analyses. We used time rather than distance to aggregate counts as it was easier to standardise in the field and provided a relevant measure of survey “effort” for mobile species, such as seabirds, that were almost always observed in flight rather than resting on the water.

### Hydroacoustic surveys

Hydroacoustic estimates of relative faunal biomass were generated using a hull-mounted Simrad EK80 echosounder operating at 3 frequencies (38, 70, and 120 kHz) with a 2 s ping rate. The acoustic settings and calibration parameters used are listed in Table J in S1 Text. Surveys consisted of a combination of nocturnal (20:00 to 06:00) and diurnal (07:00 to 18:00) transects, supplemented with more focussed nocturnal “zigzag” surveys over the summits of the Southern Seamounts (Fig 1). Acoustic data analysis was performed by developing a series of algorithms in Echoview processing software (v12+, Echoview Software Pty Ltd, Hobart) and consisted of 2 main steps: (1) data cleaning to exclude various forms of noise; (2) echo classification to assign the observed backscatter into coarse taxonomic groupings (fish, zooplankton) based on characteristic frequency responses following the methodology of Ballon and colleagues [47] (see S1 Text). For each of the final classes obtained by the discrimination algorithm, we then exported the nautical area scattering coefficient (NASC; a proxy for biomass) integrated over 500 m distance intervals for the whole water column (0 to 300 m) and stratified by depth layers (every 50 m) which formed the response variables in subsequent analyses. The fish NASC was exported at 38 kHz using a minimum threshold value of −70 dB while plankton NASC was exported at 120 kHz using a minimum threshold of −80 dB. Spatial patterns in total water column NASC of each taxon was mapped using ordinary kriging performed using the *gstat* [48] package for *R*, taking the centroid of each distance interval as the measurement point.

### Seamount upwelling and primary productivity

To provide contemporaneous estimates of water column structure and primary productivity around seamounts at the time of our biodiversity surveys, conductivity-temperature-depth (CTD) profiles (≥100 m depth) were collected at varying distances from seamount summits using a Valeport MIDAS CTD+ (*MV Extractor*) and SeaBird SBE 911+ CTD (*RRS James Clark Ross*) with integrated fluorometers for assessment of water column chlorophyll-α concentration (Fig 1B). From each profile, we then extracted the depth of the surface mixed layer (isothermal layer) along with the depth and concentration of the deep chlorophyll maximum (DCM). Fluorometers were not calibrated to local conditions and thus provided relative estimates of chlorophyll concentration only. To account for different calibration settings, the instrument used was included as a covariate in all subsequent analyses.

Seamount-induced chlorophyll enhancement (SICE) has been shown to be an ephemeral phenomenon that may not be detected in snapshots from ship-based surveys [15]. To provide a longer-term integration of primary productivity around our study seamounts, we therefore used high-resolution satellite imagery (https://resources.marine.copernicus.eu/, product: OCEANCOLOUR-GLO-CHL-L3-REP-OBSERVATIONS-009-085) to calculate 5-year (2016 to 2020) climatological mean sea surface chlorophyll (SSCHL) anomalies for all 4 × 4 km cells located within a 40 km radius of the summit of each feature. To assist with the detection of weak seamount effects, we also computed a local “Chlorophyll Enrichment Index” (CEI) for each daily scene following methods described in [24] and calculated the 5-year mean CEI for each cell. The CEI is defined as the percentage difference between the chlorophyll concentration measured at each cell and the mean value of all cells in a reference area located within a 30 to 90 km radius. For all analyses, we excluded satellite scenes with >50% cloud cover to reduce artefacts resulting from spatially uneven sampling. For analysis of seamount distance effects, we then calculated the average chlorophyll concentration and CEI in 1 km increments from the nearest seamount summit, helping to eliminate spatial autocorrelation in the raw raster values.

SICE is expected to arise due to upwelling of cool, nutrient rich waters driven by interactions between seamounts and ocean tides and currents. To explore potential upwelling effects, we analysed high-resolution time series of water temperature (10 s) and relative current velocity (1 s interval) recorded over a 600-day period by acoustic telemetry receivers (see below) moored at depths of 84 to 306 m around the summits of Grattan and Young seamounts. Time series were de-trended using loess smoothers and processed using spectral density estimation (*spectrum* function in R v3.6.1 [49]) to assess the amplitude and frequency of any periodic oscillations that might indicate diapycnal mixing.

### Testing for seamount distance effects

The influence of seamounts on all oceanographic and ecological variables measured during the study was evaluated using generalised additive (mixed) models (GAM(M)s) implemented in the package *mgcv* [50] for R v 3.6.1 [49]. To each response variable (*y*) we fit a model of the basic form:

$$g(E(y_{i}))=\beta_{0}+f_{s}({distance}_{i})+\beta_{1}x_{i}$$

Where *E*(*y*) is the expected value, *f*(*distance*) is a penalised smooth function (thin plate spline basis) of distance from the nearest seamount summit to capture any nonlinearity in the form of the relationship, *β*_1_ is the coefficient of an optional parametric term *x* (“season” in analysis of BRUV and visual transect data and “vessel” for CTD data) and *g* is a link function to map values from the response scale to the scale of linear predictor (identity in the Gaussian case and natural logarithm in the non-Gaussian). The exception was the analysis of depth-stratified hydroacoustic data where we specified a 2D tensor product smooth of distance and depth, *f*(*distance*, *depth*), to model the interacting effects of these 2 variables. To allow for variable seamount topography, including seamounts with multiple subpeaks, we defined the “summit” as the area shallower than 200 m for the Southern Seamounts and shallower than 300 m for Harris-Stewart (S1 Fig). Distance was then measured to the deployment location (BRUVs, CTDs), centroids of transect segments (hydroacoustic and visual surveys) or centroids of raster cells (SSCHL, Enrichment Index). We used distance from summit rather than bathymetry to test seamount effects because: (1) global altimetry-derived bathymetry was coarse beyond the area mapped in this study and was shown to be inaccurate for some of our features; and (2) for epipelagic species, distance from topographic features is likely to be a more important predictor of habitat use than bathymetry once seabed depths exceed >1,000 m.

Conditional distributions of the response variables were assumed to be negative binomial for overdispersed counts (BRUV and visual survey data), Tweedie for nonnegative continuous variables (hydroacoustic NASC), and Gaussian for oceanographic variables (DCM, DCM depth, ML depth, SSCHL). For continuous sampling methods (visual and hydroacoustic surveys), we included a random effect of transect to reflect the hierarchical structure of the data and, where necessary, incorporated nested autoregressive error structures to avoid overfitting associated with residual spatial and temporal autocorrelation. To determine whether seamount-distance effects differed significantly between our study features, for each response variable we initially compared 3 sets of models in which smooth function, *f*, was (1) allowed to vary by individual seamount; (2) varied by seamount depth (i.e., separate smooths for the shallow, Southern seamounts and deeper, Harris Stewart seamount); or, (3) assumed a single, global smooth for all features. Distance effects were fit as factor-smooth interactions using the “*by*” command in “*mgcv*” and the best model selected by minimization of the Akaike’s information criterion (AIC_c_). AIC is not appropriate for comparing GAMMs with non-Gaussian errors fit using penalised quasi-likelihood, so in this case we used the “*smooth_difference*” function in R package “*gratia*” [51] to compute the 95% confidence intervals (CIs) of the differences between the fitted splines at 100 regularly sampled points along the distance axis. Where 2 splines did not differ significantly along their entire lengths (i.e., 95% difference CIs included zero), we assumed that a single smooth was sufficient. Final tests of variable significance were based on Bayesian *p*-values estimated in package *mgcv* [52], as this provides a consistent approach for GAMs fit using maximum-likelihood and GAMMs fit using PQL. Full details of model fitting, selection, and checking procedures and associated R code are provided in S1 Text and S1 Code.

### Estimating seamount radius of influence

Since one of our objectives was to assess the spatial extent and magnitude of any seamount aggregating effect, wherever a significant distance effect was detected, the “*derivatives*” function in the R package g*ratia* [51] was used to identify regions of the fitted GAM(M) spline where the slope of the relationship was significantly nonzero (i.e., 95% CIs around the finite difference first derivatives did not overlap zero; see S1 Text). We defined the radius of influence (*R*) of the seamount as the furthest point at which a significant change was first detected and the magnitude of the effect as the ratio between the predicted value at the summit (i.e., *E*(*y*|*distance* = 0)) and the mean predicted value at distances > *R* (i.e., pelagic baselines). Uncertainty in the magnitude was estimated by repeatedly simulating from the posterior of the fitted GAM(M) (*n* = 1,000 simulations), recomputing the percentage change and extracting the 95% CIs. Simulations indicated that this method accurately retrieved *R* except for non-Gaussian cases where the pelagic baseline approached zero, because the “seamount effect” could not be distinguished from a monotonically increasing, linear relationship on the scale of the linear predictor. This primarily affected MaxN abundances of some individual species in BRUV surveys. In these cases, we defined R as the distance at which model predicted abundance exceeded mean MaxN from a larger set of oceanic reference BRUVs deployed throughout the Ascension Island EEZ over a similar period, or 0.05, whichever was greater. The magnitude of the seamount effect was not estimated in these cases. Custom R code used for estimating extent and magnitude of seamount effects is provided in S1 Code.

### Predator tagging

Study animals were captured opportunistically with baited handlines (sharks), by trolling lures (tuna on Southern Seamounts) or using short, vertical longlines (tuna on Harris Stewart) after which they were immobilised and winched to deck level using a custom tagging sling. Throughout all tagging procedures, a saltwater hose inserted into the mouth was used to continuously irrigated the gills and a wet cloth was placed over the eyes to minimise distress. Galapagos and silky sharks were fitted with internal acoustic fish tags (Vemco V16-6x, Bedford, Nova Scotia, Canada; dimensions: 16 × 95 mm) implanted into the abdominal cavity through a small incision made anterior to the anal fin and offset from the midline. Surgical implements and the insertion site were first sterilised with a Betadine solution and the wound was closed with surgical sutures. For a subset of individuals, Fastloc GPS-enabled Argos transmitters (SPOT-F-338, Wildlife Computers, Redmond, Washington, United States of America) were also mounted to the dorsal fin using plastic hex bolts and stainless-steel lock nuts such that the base of antenna was level with the apex of the fin. All tuna were equipped with pop-up satellite archival tags (MiniPAT, Wildlife Computers) that were secured through the dorsal pterygiophores using titanium darts and monofilament tethers as described previously [53]. Tags were programmed to release automatically after periods of 180 to 360 days or following >72 h at constant depth (±3 m). Complementary acoustic tagging of tuna was trialled but abandoned as the exceptionally high density of sharks was assessed to severely compromise the survival of fish released following surgery.

### Acoustic array design

Acoustically tagged sharks were tracked using fixed receiver arrays (Vemco VR2AR-69khz) established on the summits of the Grattan and Young seamounts between 1st and 3rd June 2017 (*n* = 7 per seamount). Receivers were deployed encircling the summit plateau of Grattan and on prominent peaks and ridges of the more topographically complex Young seamount (S1 Fig). Mean (± SD) seabed depth at deployment locations was 161 ± 64 m on Grattan (range = 131 to 306 m) and 158 ± 48 m on Young (range = 84 to 202 m). Average minimum spacing between receivers was 1.36 ± 0.9 km on Grattan (range = 0.88 to 3.43 km) and 1.75 ± 0.3 km on Young (range = 1.48 to 2.17 km). Receivers were tethered to a ~100 kg anchor and suspended 10 m above the seabed attached to a syntactic foam buoy (HBF-13-750, DeepWater Buoyancy, Maine, USA) providing approximately 10 kg of lift to allow subsequent retrieval at the surface. Range tests were conducted on a subset of receivers (*n* = 5) using a matching acoustic tag programmed with a 10-s ping rate and suspended approximately 10 m below a slowly drifting vessel whose position was continuously logged using GPS. Binomial GAMMs were then used to model the relationship between detection probability (proportion of expected detections received per 180 s time bin) and distance from the receiver (including horizontal and vertical displacement from the test tag). Following Scherrer and colleagues [54], we defined the average maximum detection radius (AMDR) as the point at which mean predicted detection probability dropped below 5%, estimated as 839 m across the 5 sites tested. Receivers were recovered by triggering the integrated acoustic release mechanism with a Vemco VHTx transponding hydrophone: once in January 2018 to service and download data and again in February 2019 when the study was terminated.

### Acoustic telemetry data analysis

Detection time series of acoustically tagged sharks were analysed at 2 levels to describe seamount interactions at different temporal and spatial scales. To quantify overall residency within the Southern Seamounts, we first calculated the proportion of total tracking days that an individual was detected at least once on any receiver in the array and used quasibinomial GLMs to test effects of species, sex, and size class on the resulting residency index (RI). Thin plate spline regressions implemented in package *mgcv* were also used to model how the proportion of tagged individuals still present on the array (i.e., >1 detection day) varied over time for each species. To allow for behavioural equilibration after tagging, each individual’s track was deemed to have started following first detection on summit receiver arrays on at least 2 consecutive days. Six individuals were never detected following release, and since their fate is unknown (e.g., mortality, tag failure/ejection, dispersal), they are excluded from subsequent analysis.

For the second level of the analysis, we considered only those periods when individuals were present on the array (defined as ≥14 consecutive days with ≥1 detection day^-1^). To explore diel variability in usage of seamounts, we first calculated individual hourly RIs (proportion of hourly bins in which an individual was detected at least once on the array) and analysed trends using a quasibinomial GAMM including a cubic cyclic smoothing spline of hour (to ensure ends of the spline meet) and a random effect of individual. Finer scale space use was also examined by calculating the proportion of an individual’s total detections that were recorded on each receiver in the array during each diel phase (day/night). Mean proportions and bootstrapped confidence intervals were then calculated for each species.

### Satellite telemetry data analysis

Positional data from SPOTs and PSATs were processed using tag-specific state-space models (SSMs) to estimate most probable locations at regular 12-h intervals along with their associated posterior probability distributions. SPOT data were analysed using continuous time correlated walk (CTCRW) models implemented in the R package *crawl* [55], whereas PSAT data were processed using the manufacturer’s proprietary GPE3 model following methods described by Richardson and colleagues [53] (with a travel speed standard deviation of 1.4 FL s^-1^). To avoid excessive interpolation associated with sparse data and long transmission gaps, SPOT tracks were first cut into segments with ≤14 days between successive locations and CTCRW models fit only to those segments with >10 locations. To investigate predator site fidelity and space use around seamounts, for each tagged individual and population (i.e., species-seamount cohort), we first derived an overall probability distribution describing the likelihood that a given area was used by merging the individual 12-hourly probability density surfaces on a common grid using a weighted average that gives greater influence to animals with longer deployment periods (see [53] for details). For each individual, we also calculated the distance travelled from their original tagging seamount at each interpolated point along their posterior mean track and computed the 95% CIs using 1,000 random draws from the posterior distribution of each location. Since location errors associated with PSAT geolocations are typically large, high-quality Argos positions (classes 1–3) at the point of tag release often provide the only confident estimate of position. To assess whether tagged tuna were located closer to seamounts at this release point than would be expected by chance, we simulated 10,000 correlated random walks with the same duration and movement parameters (i.e., step length and turning angle distributions) as the observed tracks (see S1 Text and S2 Code for details). The proportion of simulations that ended closer to seamounts than each observed track was then used as a test of the null hypothesis that an individual showed no site fidelity (α = 0.05).

## Supporting information

S1 Text Supplementary results and methods, including Tables A–K. (DOCX)

S1 Fig Bathymetry and oceanography of study seamounts. (A) High-resolution (25 m) multibeam bathymetry of the Harris Stewart, Grattan and Young seamounts. Triangle markers denote the shallowest point of each feature. (B) Bathymetric profiles along transect lines (x → y) in (A) overlaid on mean water column chlorophyll A concentration in 5 m depth increments calculated from all CTD deployments made over each feature (May–June 2017). The mean depth of the surface mixed layer (65–67 m) in CTD deployments and the approximate limit of the photic zone (200 m) are also shown for reference. (C) Periodograms of 595-day temperature and current strength time series recorded by 14 acoustic telemetry receivers deployed on the summits of the Grattan and Young seamounts (positions marked in (A)). Plots show the mean spectral density averaged across all receivers for the 0–26 h frequency range. Note the dominant 12.4-h periodicity in both series corresponding to the frequency of the principle semi-diurnal lunar tide. The data underlying this figure can be found in S1 Data (A) and S5 Data (B and C).
(TIF)

S2 Fig Locations of “oceanic” BRUV surveys (i.e., > 50 km from any island or seamount) conducted haphazardly throughout the Ascension Island EEZ in January–February 2017 and 2018. The 50 km feature buffers are marked in white. Data from these surveys was used to provide additional estimates of baseline abundances of pelagic predators in addition to those derived from radial sampling around seamounts (see Table B in S1 Text). The data underlying this figure can be found in S2 Data.
(TIF)

S3 Fig Variation in relative abundance of predatory fish and sharks observed in BRUVs surveys as a function of distance from Ascension Island’s outlying seamounts. Relative abundance is expressed as the maximum number of individuals of a given species observed in a single video frame (MaxN). Only data from species observed in >5 surveys are presented. Explanations of other plotting elements follow Fig 2. The data underlying this figure can be found in S2 Data. Illustrations: Marc Dando (sharks) and Diane Rome Peebles (fish).
(TIF)

S4 Fig Variation in relative abundance of seabirds observed in vessel-based visual transects as a function of distance from Ascension Island’s outlying seamounts. Relative abundance is expressed as the number of individuals observed in a 300 m belt transect centred on the vessel per 5 min sampling interval. Only data from species observed in >5 sampling intervals are presented. Solid trend lines and shaded envelopes are fitted smooths and associated 95% CIs from negative binomial GAMMs with a random effect of transect id. Illustrations: *F*. *aquila* and *O*. *fuscatus*, Peter Harrison; *O*. *Castro* and *Procellaridae* sourced via Wikimedia under a Creative Commons license. The data underlying this figure can be found in S3 Data.
(TIF)

S5 Fig Variation in hydroacoustic nautical area scattering coefficients (NASC) of zooplankton and pelagic with distance from the summits of Ascension Island’s outlying seamounts. Plotted points are total water column NASC from 0–300 m depth, integrated over 500 m distance sampling units and trend lines are predicted means from fitted GAMs and their associated 95% confidence intervals (see Table E in S1 Text). Fitted smooths were initially allowed to vary for the 2 Southern Seamounts but were only retained for pelagic fish where trends were significantly non-overlapping. For zooplankton, a single global smoother is presented. The data underlying this figure can be found in S4 Data. Illustrations: Creative Commons via Wikimedia.
(TIF)

S6 Fig Localised hotspots of zooplankton and pelagic fish biomass over the summits of Ascension Island’s Southern Seamounts based on hydroacoustic surveys conducted in May–June 2017. Heatmaps were derived using ordinary kriging of total water column (0–300 m) nautical area scatterning coefficients (NASCs) integrated over 500 m distance sampling units. To avoid over-interpolation, kriging was limited to an area within 800 m of the survey transect. Arrow vectors in each plot represent the mean geostropic current flows over each seamount during hydroacoustic surveys based on time-matched satellite altimery data (source: Copernicus Marine Service GlobCurrent, dataset MULTOBS_GLO_PHY_MYNRT_015_003). The data underying this figure can be found in S4 Data. Illustrations: Creative Commons via Wikimedia.
(TIF)

S7 Fig Localised hotspots of zooplankton and pelagic fish biomass over the summits of Ascension Island’s Southern Seamounts based on hydroacoustic surveys conducted in May–June 2017. Heatmaps were derived using ordinary kriging of total water column (0–300 m) nautical area scatterning coefficients (NASCs) integrated over 500 m distance sampling units. To avoid over-interpolation, kriging was limited to an area within 800 m of the survey transect. Arrow vectors in each plot represent the mean geostropic current flows over each seamount during hydroacoustic surveys based on time-matched satellite altimery data (source: Copernicus Marine Service GlobCurrent, dataset MULTOBS_GLO_PHY_MYNRT_015_003). The data underying this figure can be found in S4 Data. Illustrations: Creative Commons via Wikimedia.
(TIF)

S8 Fig Variation in biomass and abundance of mid-trophic epipelagic forage fish as a function of distance from seamount summits. **(A)** Relative biomass of epipelgic forage fish observed in shallow (10 m) pelagic BRUV deployments. Biomass is expressed as the maximum biomass of forage fish (see Table B in S1 Text) observed in a single video frame, based on photogrammetric fork length measurements of individual animals converted using species-specific length–weight relationships (see Methods). Broken lines represent regional oceanic baselines from a reference set of 56 BRUVs surveys conducted >50 km from any seamount or island over a similar period (S2 Fig). (B) Relative abundance of flying fish (*Exocoetidae* sp.) observed in vessel-based visual transects. Abundance (N) is expressed as the number of individuals observed in a 300 m belt transect centred on the vessel per 5 min sampling interval. Solid trend lines and shaded envelopes are fitted smooths and associated 95% confidence intervals from Tweedie GAMs for biomass and negative binomial GAMMs with a random effect of transect id for visual transects. The data underlying this figure can be found in S2 Data (A) and S3 Data (B). Illustrations: Creative Commons via Wikimedia.
(TIF)

S9 Fig Pluriannual mean (2016–2020) sea surface chlorophyll A concentration and chlorophyll enrichment indices (CEI) in the vicinity of Ascension Island’s outlying seamounts derived from 4 × 4 km resolution satellite data. CEIs are percentage deviations from a moving average of all cells located within 30–90 km of the focal cell and help strengthen any local seamount signal by removing mesoscale gradients (see Methods). In each map, arrow vectors show the average current strength and direction across the study period based on satellite altimetry data (source: Copernicus Marine Service, dataset MULTIOBS_GLO_PHY_REP_015_004). Plots below each map show how mean chlorophyll concentrations and enrichment indices vary as a function of distance from the nearest seamount summit. In each case, plotted points are the mean cell values calculated in 1 km increments radiating out from the summit and trend lines are predictions from fitted GAMs and associated 95% confidence intervals (see Table G in S1 Text). The data underlying this figure can be found in S5 Data.
(TIF)

S10 Fig Abacus plot showing detection histories of 15 juvenile Galapagos sharks (*Carcharhinus galapagensis*) and 14 silky sharks (*Carcharhinus falciformis*) tagged with internal acoustic tags on Ascension Island’s Southern Seamounts in June 2017. Symbols are shaded according to the seamount on which detections occurred and are scaled according to the number of hourly bins on each day in which an animal was detected at least once on summit receiver arrays (S1 Fig). Note the periodic movements between seamounts observed in some individuals. The data underlying this figure can be found in S6 Data. Illustrations: Marc Dando.
(TIF)

S1 Data Multibeam bathymetry data of the Grattan, Young and Harris Stewart Seamounts collected by the RRS James Clarke Ross in May–June 2017. (ZIP)

S2 Data Total biomass, species richness, and abundance (Max N) data from baited remote underwater video surveys conducted around Ascension Island’s seamounts and adjacent oceanic waters. (XLSX)

S3 Data Nautical area scattering coefficients of fish and zooplankton from hydroacoustic surveys conducted around Ascension Island’s seamounts in May–June 2017, including total water column (0–300 m) and disaggregated by depth strata. (XLSX)

S4 Data Abundances of seabirds and flying fish recorded in vessel based visual transects conducted around Ascension Island’s seamounts in May–June 2017. (XLSX)

S5 Data Oceanographic data collected around Ascension Island’s outlying seamounts including CTD profiles and derived metrics, log data from Vemco VR2AR acoustic receivers, and mean chlorophyll enrichment indices and sea surface chlorophyll concentrations derived from satellite data (see Methods). (XLSX)

S6 Data Satellite and acoustic tracking data for pelagic predators (sharks and tuna species) tagged on Ascension Island’s seamounts, along with associated metadata. (XLSX)

S1 Code Sample R code detailing procedures used for analysing seamount distance and enrichment effects. (R)

S2 Code Custom R code used for analysing pop up satellite archival tag data, including simulation-based approaches for assessing fidelity to seamounts. (R)

## Abbreviations

AIC: Akaike’s information criterion
AMDR: average maximum detection radius
BRUV: baited remote underwater video
CEI: chlorophyll enrichment index
CI: confidence interval
CTCRW: continuous time correlated walk
CTD: conductivity-temperature-depth
DCM: deep chlorophyll maximum
EEZ: exclusive economic zone
MPA: marine protected area
NASC: nautical area scattering coefficient
PSAT: pop-up satellite archival tag
RI: residency index
SICE: seamount-induced chlorophyll enrichment
SSCHL: sea surface chlorophyll
SSM: state-space model
