# Supplementary material for: Shallow seamounts are “oases” and activity hubs for pelagic predators in a large-scale marine reserve
Source: PLoS Biol. 2025 Feb 4;23(2):e3003016. doi: 10.1371/journal.pbio.3003016 (PMC11828362; doi:10.1371/journal.pbio.3003016)
Supplement: S1 Text — (DOCX) [file pbio.3003016.s001.docx]

**SUPPORTING INFORMATION:**

**SHALLOW SEAMOUNTS ARE ‘OASES’ AND ACTIVITY HUBS FOR PELAGIC PREDATORS IN A LARGE-SCALE MARINE RESERVE**

Sam B. Weber, Andrew J. Richardson, Christopher D. H. Thompson, Judith Brown, Fabio Campanella, Brendan J. Godley, Nigel E. Hussey, Jessica J. Meeuwig, Paul Rose, Nicola Weber, Matthew J. Witt, Annette C. Broderick

**CONTENTS**

[**Supplementary Results**](#AppendixS1)

**Table A**

**Table B
Table C**

**Table D**

**Table E**

**Table F**

**Table G**

**Table H**

**Table I**

[**Supplementary Methods**](#AppendixS1)

**Table J**

**Table K**

**SUPPLEMENTARY RESULTS**

**Table A.** Physical characteristics of the study seamounts

| Seamount | Minimum depth (m) | **Area (km^2^)** | |  | **Distance from (km)** | |
| --- | --- | --- | --- | --- | --- | --- |
|  |  | < 200 m | < 500 m |  | Ascension Is. | Mid-Atlantic Ridge |
| Grattan | 101 | 6.1 | 27.5 |  | 256.1 | 39.3 |
| Young | 77.3 | 3.4 | 31.3 |  | 314.5 | 117.7 |
| Harris-Stewart | 265.5 | 0 | 41.2 |  | 295.2 | 396.3 |

**Table B.** Maximum abundance (MaxN) and probability of occurrence (P) of pelagic fish, shark and cetacean species recorded during 59 mid-water baited remote underwater video (BRUV) surveys conducted within 40 km of the Harris Stewart and Southern Seamounts in May–June 2017. MaxN is the maximum number of individuals simultaneously recorded in any survey and P is the proportion of deployments in which at least one individual was detected. For comparison, summary statistics for 56 comparable BRUV surveys conducted in oceanic waters > 50 km from islands or seamounts in January–February 2017 and 2018 are also shown (S2 Fig).

|  | | | |  | **Max N** | | | **P** | | |
| --- | --- | --- | --- | --- | --- | --- | --- | --- | --- | --- |
|  | **Family** | **Species** |  | **Functional Group** | **Harris** | **Southern** | **Oceanic** | **Harris** | **Southern** | **Oceanic** |
|  | Delphinidae | *Stenella sp* |  | Dolphins | 1 | 0 | 0 | 0.07 | 0.00 | 0.00 |
| **Elasmobranchs** | Carcharhinidae | *Carcharhinus falciformis* | (Silky shark) | Sharks | 0 | 14 | 0 | 0.00 | 0.45 | 0.00 |
|  |  | *Carcharhinus galapagensis* | (Galapagos shark) | Sharks | 0 | 22 | 0 | 0.00 | 0.25 | 0.00 |
|  |  | *Carcharhinus longimanus* | (Oceanic whitetip) | Sharks | 1 | 0 | 0 | 0.07 | 0.00 | 0.00 |
|  |  | *Prionace glauca* | (Blue shark) | Sharks | 1 | 2 | 2 | 0.40 | 0.18 | 0.25 |
|  | Lamnidae | *Isurus oxyrinchus* | (Shortfin mako) | Sharks | 0 | 1 | 1 | 0.00 | 0.02 | 0.04 |
|  | Sphyrnidae | *Sphyrna zygaena* | (Smooth hammerhead) | Sharks | 1 | 1 | 1 | 0.07 | 0.02 | 0.02 |
| **Teleost** | Balistidae | *Balistes capriscus* | (Grey triggerfish) | Adult other | 0 | 1 | 0 | 0.00 | 0.02 | 0.00 |
|  |  | *Canthidermis maculata* | (Rough triggerfish) | Adult other | 0 | 1 | 0 | 0.00 | 0.05 | 0.00 |
|  | Carangidae | *Carangidae sp* |  | Forage fishes | 0 | 1 | 1 | 0.00 | 0.02 | 0.05 |
|  |  | *Caranx crysos* | (Blue runner) | Predatory teleosts | 1 | 0 | 0 | 0.07 | 0.00 | 0.00 |
|  |  | *Caranx hippos* | (Crevalle jack) | Forage fishes | 0 | 1 | 1 | 0.00 | 0.02 | 0.02 |
|  |  | *Decapterus sp* | (Mackerel scad) | Forage fishes | 0 | 1 | 1 | 0.00 | 0.02 | 0.02 |
|  |  | *Elagatis bipinnulata* | (Rainbow runner) | Predatory teleosts | 0 | 21 | 28 | 0.00 | 0.27 | 0.02 |
|  |  | *Naucrates ductor* | (Pilot fish) | Forage fishes | 1 | 2 | 4 | 0.07 | 0.14 | 0.27 |
|  | Coryphaenidae | *Coryphaena equiselis* | (Pompano dolphinfish) | Predatory teleosts | 19 | 5 | 15 | 0.13 | 0.07 | 0.14 |
|  |  | *Coryphaena hippurus* | (Common dolphinfish) | Predatory teleosts | 3 | 5 | 7 | 0.13 | 0.07 | 0.09 |
|  | Echeneidae | *Echeneis naucrates* | (Live sharksucker) | Epibionts | 1 | 3 | 1 | 0.07 | 0.11 | 0.14 |
|  |  | *Remora remora* | (Common remora) | Epibionts | 2 | 6 | 8 | 0.33 | 0.52 | 0.30 |
|  | Istiophoridae | *Istiophorus albicans* | (Atlantic sailfish) | Predatory teleosts | 1 | 1 | 1 | 0.07 | 0.18 | 0.02 |
|  |  | *Makaira nigricans* | (Atlantic blue marlin) | Predatory teleosts | 0 | 1 | 1 | 0.00 | 0.09 | 0.07 |
|  | Monacanthidae | *Aluterus scriptus* | (Scrawled filefish) | Forage fishes | 1 | 1 | 2 | 0.07 | 0.05 | 0.05 |
|  |  | *Cantherhines macrocerus* | (Whitespotted filefish) | Forage fishes | 1 | 0 | 1 | 0.13 | 0.00 | 0.05 |
|  | Nomeidae | *Psenes sp* | (Driftfish) | Forage fishes | 15 | 7 | 30 | 0.80 | 0.77 | 0.93 |
|  | Scombridae | *Acanthocybium solandri* | (Wahoo) | Predatory teleosts | 2 | 13 | 2 | 0.13 | 0.18 | 0.07 |
|  |  | *Katsuwonus pelamis* | (Skipjack tuna) | Predatory teleosts | 0 | 159 | 0 | 0.00 | 0.05 | 0.00 |
|  |  | *Scombridae sp* |  | Forage fishes | 0 | 32 | 2 | 0.00 | 0.05 | 0.02 |
|  |  | *Thunnus albacares* | (Yellowfin tuna) | Predatory teleosts | 0 | 94 | 49 | 0.00 | 0.20 | 0.05 |
|  |  | *Thunnus obesus* | (Bigeye tuna) | Predatory teleosts | 0 | 1 | 0 | 0.00 | 0.02 | 0.00 |
|  |  |  |  |  | Total deployments: | | | (n = 15) | (n = 44) | (n = 56) |

**Table C. Results of negative binomial GAMs fit to estimated total biomass, species richness and MaxN abundance of large-bodied pelagic predators observed in BRUVS surveys around Ascension islands outlying seamounts.** Results are presented as the estimated degrees of freedom (e.d.f), Wald statistics and *p*-values of a smooth function relating response variables to distance from the nearest seamount summit, $f$(distance). Where a significant effect was detected, the seamount radius of influence (R) was also estimated as the maximum distance from the summit at which fitted mean values significantly exceeded a regional oceanic baseline (see Methods). Data for the Southern Seamounts were pooled based on initial AIC-based model comparisons which favoured a common smoother to describe trends in biomass and species richness around these adjacent, shallow features. Observations of individual species were generally too infrequent to robustly model seamount-specific trends in MaxN. We therefore adopted the same clustering used for overall biomass and richness, pooling data for the Southern Seamounts, and limited the analysis of abundance trends to species encountered in > 6 surveys (Table B).

| **Response** | **Taxa** | $\boldsymbol{f}$**(distance)** | | | **R (km)** | |  |
| --- | --- | --- | --- | --- | --- | --- | --- |
|  |  | e.d.f. | $\chi^{2}$ | *p* |  |  |  |
|  | **Southern Seamounts (shallow)** | | | |  |  | |
| Biomass | All pelagic predators | 2.95 | 15.3 | **<0.001** | 4.0 | |  |
| Biomass | Sharks | 3.15 | 13.6 | **<0.001** | 5.4 | |  |
| Biomass | Predatory teleosts | 1.82 | 16.7 | **<0.001** | 3.6 | |  |
| Biomass | Forage fish | 1.00 | 1.26 | 0.27 | - | |  |
| Species Richness | All pelagic predators | 2.78 | 39.1 | **<0.001** | 5.3 | |  |
| MaxN | *Elagatis bipinnultata* | 1.00 | 18.6 | **<0.001** | 1.2 | |  |
| MaxN | *Carcharhinus falciformis* | 2.45 | 34.6 | **<0.001** | 5.0 | |  |
| MaxN | *Carcharhinus galapagensis* | 1.00 | 11.8 | **<0.001** | 2.1 | |  |
| MaxN | *Acanthocybium solandri* | 1.00 | 4.37 | **0.037** | 5.0 | |  |
| MaxN | *Thunnus albacares* | 1.00 | 9.76 | **0.002** | 1.5 | |  |
| MaxN | *Coryphaena spp.* | 1.00 | 0.13 | 0.72 | - | |  |
| MaxN | *Istiophorus albicans* | 1.00 | 0.38 | 0.54 | - | |  |
| MaxN | *Prionace glauca* | 1.00 | 0.14 | 0.71 | - | |  |
|  | **Harris Stewart Seamount (deep)** | | | |  |  | |
| Biomass | All pelagic predators | 1.00 | 0.28 | 0.61 | - | |  |
| Biomass | Sharks | 1.00 | 0.11 | 0.75 | - | |  |
| Biomass | Predatory teleosts | 1.00 | 10.5 | **0.006** | - | |  |
| Biomass | Forage fish | 3.57 | 10.6 | **0.002** | - | |  |
| Species Richness | All pelagic predators | 1.00 | 0.33 | 0.57 | - | |  |
| MaxN | *Prionace glauca* | 1.00 | 0.15 | 0.70 | - | |  |

**Table D. Total counts (N) and probability of occurrence (P) of seabirds and flying fish in vessel-based visual surveys carried out around Ascension Island’s outlying seamounts.** N is the total count summed across all transects around a given feature and the P is the proportion of 5-minute sampling intervals in which at least one individual was detected.

|  | | | | **N** | | **P** | |
| --- | --- | --- | --- | --- | --- | --- | --- |
|  | **Family** | **Species** |  | **Harris** | **Southern** | **Harris** | **Southern** |
| **Fish** | *Exocoetidae* | *Exocoetus spp.* | (Flying fish) | 1238 | 1695 | 0.65 | 0.50 |
| **Seabirds** | *Fregatidae* | *Fregata aquila* | (Ascension frigatebird) | 0 | 10 | 0.00 | 0.03 |
|  | *Laridae* | *Gygis alba* | (White tern) | 0 | 3 | 0.00 | 0.004 |
|  |  | *Onychoprion fuscatus* | (Sooty tern) | 2 | 97 | 0.005 | 0.05 |
|  | *Procellaridae* | *Procellaridae sp.* | (Shearwaters) | 0 | 18 | 0 | 0.06 |
|  | *Hydrobatidae* | *Oceanodroma castro* | (Band-rumped storm petrel) | 6 | 30 | 0.02 | 0.10 |
|  | *Sulidae* | *Sula dactylatra* | (Masked booby) | 1 | 0 | 0.005 | 0 |
|  |  | *Sula leucogaster* | (Brown booby) | 0 | 1 | 0.004 | 0.09 |
|  |  |  |  | Total sampling units: | | (199) | (267) |

**Table E. Results of negative binomial GAMMs fitted to species counts from vessel-based visual surveys.** Only species encountered in ≥ 6 surveys were included in the analysis. In each case, results are presented as the estimated degrees of freedom (e.d.f), Wald statistics and *p*-values of a smooth function relating species counts to distance from the nearest seamount summit. No seabird species were observed sufficiently frequently around the Harris Stewart Seamount to test distance effects.

| **Species** |  | $\boldsymbol{f}$**(distance)** | | |
| --- | --- | --- | --- | --- |
|  |  | e.d.f. | $\chi^{2}$ | *p* |
| **Southern seamounts** | | | | |
| *Fregata aquila* | (Ascension frigatebird) | 1.00 | 4.43 | **0.035** |
| *Oceanodroma castro* | (Storm petrel) | 1.00 | 0.01 | 0.91 |
| *Onychoprion fuscatus* | (Sooty tern) | 1.00 | 11.0 | **<0.001** |
| *Procellariidae sp.* | (Shearwaters) | 1.00 | 0.11 | 0.74 |
| *Excoetidae sp.* | (Flying fish) | 1.00 | 4.02 | **0.045** |
| **Harris Stewart Seamount** | | | | |
| *Excoetidae sp.* | (Flying fish) | 1.00 | 0.36 | 0.55 |

**Table F.** **Results of Tweedie GAMMs fit to total watercolumn (0-300m) nautical area scattering coefficients (NASC) of zooplankton and fish measured during nocturnal and dirunal hydroacoustic surveys around Ascension Island’s outlying seamounts.** For each taxa and diel phase, NASC was modelled as a smooth function of distance from the nearest seamount, $f$(distance), and the null hypothesis that $f=0$ was tested using *p*-values based on Wald-like tests. The estimated degrees of freedom (e.d.f) describes the wiggliness of the fitted smoother, where e.d.f = 1 is equivalent to a linear relationship on the scale of the linear predictor. All models included a random effect of transect with a nested first order autoregressive process to account for serial non-independence between consecutive distance sampling units. Where a significant effect was detected, the seamount radius of influence (R) was also estimated as the maximum distance from the summit where NASC began to significantly increase based on the derivatives of the fitted smooths.

| **Taxon** | **Period** | **Seamount(s)** | $\boldsymbol{f}$**(distance)** | | | **R (km)** |
| --- | --- | --- | --- | --- | --- | --- |
|  |  |  | e.d.f. | *F* | *p* |  |
| **Southern Seamounts (shallow)** | | | | | | |
| Zooplankton | Day | Southern | 1 | 0.51 | 0.47 | - |
| Zooplankton | Night | Southern | 5.4 | 10.5 | **<0.001** | 3.1 |
| Fish | Day | Southern: Grattan | 4.2 | 38.4 | **<0.001** | 4.9 |
|  |  | Southern: Young | 1.0 | 10.2 | **0.002** | - |
| Fish | Night | Southern: Grattan | 4.4 | 11.9 | **<0.001** | 3.8 |
|  |  | Southern: Young | 6.4 | 8.8 | **<0.001** | 2.1 |
| **Harris Stewart Seamount (deep)** | | | | | | |
| Zooplankton | Night | Harris Stewart | 1.0 | 1.7 | 0.2 |  |
| Fish | Night | Harris Stewart | 2.37 | 8.1 | **<0.001^*^** | 7.3* |
| *Significance is driven by a single influential measurement (Figure S5) and R is likely unreliable. | | | | | | |

**Table G.** **Summary output of GAMs fit to oceanographic variables extracted from CTD profiles collected around Ascension Island’s outlying seamounts along with 5-year mean sea surface chlorophyll A concentrations (SSCHLA) and chlorophyll enrichment indices (CEI) derived from satellite data**. Explanatory variables included a smooth function of distance from the nearest seamount summit and survey vessel (factor) to control for different CTD calibration settings. Results are presented as test statistics and *p*-values from Wald-like tests, along with the estimated degrees of freedom (e.d.f.) for smooth terms and estimated regression coefficients for parametric terms.

| **Variable** | **Seamount(s)** | **Distance from summit** | | | **Vessel** | | |
| --- | --- | --- | --- | --- | --- | --- | --- |
|  |  | e.d.f. | *F* | *p* | est ±SD | *t* | *p* |
| Deep chlorophyll maximum (DCM) | Southern | 1 | 0.07 | 0.79 | 0.08 ± 0.01 | 10.3 | <0.001 |
|  | Harris Stewart | 1 | 3.18 | 0.11 | 0.12 ± 0.02 | 6.19 | <0.001 |
| DCM depth | Southern | 1 | 0.17 | 0.68 | 3.27 ± 2.11 | 1.5 | 0.13 |
|  | Harris Stewart | 1 | 1.29 | 0.28 | -3.05 ± 9.42 | -0.32 | 0.75 |
| Mixed layer depth | Southern | 1 | 1.37 | 0.25 | 1.48 ± 2.32 | 0.64 | 0.52 |
|  | Harris Stewart | 1 | 0.46 | 0.51 | -12.6 ± 6.76 | -1.87 | 0.09 |
| Surface Chl-a | Southern | 1 | 2.17 | 0.15 | NA | | |
|  | Harris Stewart | 1 | 1.52 | 0.23 | NA | | |
| CEI | Southern: Young | 2.78 | 11.1 | <0.001 | NA | | |
|  | Southern: Grattan | 1.41 | 3.72 | 0.06 | NA | | |
|  | Harris Stewart | 2.25 | 15.5 | <0.001 | NA | | |

**Table H.** Summary of acoustic and Argos-GPS (SPOT) telemetry tag deployments on 36 Galapagos and silky sharks at the Grattan and Young Seamounts. The residency index is calculated as the proportion of total tracking days on which acoustically-tagged animals were detected at least once on summit receiver arrays. The number of filtered SPOT locations received from double tagged animals is also shown.

| ID | Seamount | Tag date | Sex | Length (cm) | | Tag type | | Residency index | SPOT locations* |
| --- | --- | --- | --- | --- | --- | --- | --- | --- | --- |
|  |  |  |  | Total | Fork | Acoustic | SPOT |  |  |
| Galapagos shark (*Carcharhinus galapagensis*) | | | | | | | | | |
| GAL23 | Young | 30 May 2017 | M | 170 | 146 | TRUE | TRUE | 1.00 | 3 |
| GAL24 | Young | 30 May 2017 | M | 163 | 129 | TRUE | TRUE | 0.95 | 0 |
| GAL25 | Young | 31 May 2017 | M | 145 | 118 | TRUE | TRUE | 0.89 | 0 |
| GAL26 | Young | 31 May 2017 | F | 161 | 125 | TRUE | TRUE | 0.97 | 18 |
| GAL27 | Young | 31 May 2017 | M | 204 | 164 | TRUE | TRUE | 0.98 | 4 |
| GAL28 | Young | 31 May 2017 | M | 170 | 131 | TRUE | TRUE | 0.89 | 0 |
| GAL29 | Grattan | 01 Jun 2017 | F | 168 | 123 | TRUE | TRUE | 0 | 0 |
| GAL30 | Grattan | 01 Jun 2017 | M | 245 | 194 | TRUE | TRUE | 0.96 | 3 |
| GAL31 | Grattan | 01 Jun 2017 | M | 120 | 96 | TRUE | FALSE | 0 | - |
| GAL32 | Grattan | 02 Jun 2017 | M | 161 | 135 | TRUE | TRUE | 1.00 | 0 |
| GAL33 | Grattan | 02 Jun 2017 | M | 130 | 107 | TRUE | FALSE | 0.97 | - |
| GAL34 | Grattan | 02 Jun 2017 | M | 157 | 126 | TRUE | FALSE | 1.00 | - |
| GAL35 | Grattan | 02 Jun 2017 | M | 156 | 124 | TRUE | FALSE | 1.00 | - |
| GAL36 | Grattan | 02 Jun 2017 | M | 148 | 112 | TRUE | FALSE | 1.00 | - |
| GAL37 | Grattan | 02 Jun 2017 | M | 126 | 108 | TRUE | FALSE | 1.00 | - |
| GAL38 | Grattan | 02 Jun 2017 | M | 155 | 124 | TRUE | FALSE | 1.00 | - |
| GAL39 | Grattan | 02 Jun 2017 | M | 135 | 105 | TRUE | FALSE | 1.00 | - |
| Silky shark (*Carcharhinus falciformis*) | | | | | | | | | |
| SILK02 | Young | 30 May 2017 | M | 142 | 115 | TRUE | FALSE | 0.002 | - |
| SILK03 | Young | 30 May 2017 | M | 180 | 143 | TRUE | FALSE | 0.76 | - |
| SILK04 | Young | 30 May 2017 | M | 136 | 115 | TRUE | TRUE | 0 | 0 |
| SILK05 | Young | 31 May 2017 | M | 175 | 137 | TRUE | TRUE | 0.11 | 15 |
| SILK06 | Young | 01 Jun 2017 | F | 168 | 138 | TRUE | FALSE | 0.99 | - |
| SILK07 | Young | 01 Jun 2017 | M | 153 | 123 | TRUE | FALSE | 0.34 | - |
| SILK08 | Young | 01 Jun 2017 | M | 160 | 123 | TRUE | FALSE | 1.00 | - |
| SILK09 | Young | 01 Jun 2017 | M | 147 | 121 | TRUE | FALSE | 0.97 | - |
| SILK10 | Grattan | 01 Jun 2017 | F | 156 | 122 | TRUE | FALSE | 0.07 | - |
| SILK11 | Grattan | 01 Jun 2017 | F | 181 | 152 | TRUE | TRUE | 0.04 | 14 |
| SILK12 | Grattan | 02 Jun 2017 | M | 123 | 102 | TRUE | FALSE | 0.90 | - |
| SILK13 | Grattan | 02 Jun 2017 | M | 141 | 118 | TRUE | TRUE | 0.09 | 9 |
| SILK14 | Grattan | 02 Jun 2017 | M | 126 | 101 | TRUE | FALSE | 0 | - |
| SILK15 | Grattan | 02 Jun 2017 | M | 167 | 135 | TRUE | TRUE | 1 | 28 |
| SILK16 | Grattan | 02 Jun 2017 | F | 155 | 120 | TRUE | TRUE | 0.85 | 13 |
| SILK17 | Grattan | 02 Jun 2017 | M | 200 | - | TRUE | TRUE | 0.06 | 17 |
| SILK18 | Grattan | 02 Jun 2017 | F | 178 | 144 | TRUE | TRUE | 0 | 0 |
| SILK19 | Grattan | 02 Jun 2017 | M | 148 | 119 | TRUE | TRUE | 0 | 0 |
| SILK20 | Grattan | 02 Jun 2017 | M | 141 | 116 | FALSE | TRUE | - | 41 |

* Only locations that pass speed and Argos location class filtering shown

**Table I. Summary of pop-up satellite archival tag deployments on 22 yellowfin and bigeye tuna at the Grattan and Young Seamounts.** Tracking duration is the interval from deployment to tag release, minus any period floating at the surface or at constant depth that triggered release. Displacement is the distance from the nearest seamount summit to the first high quality Argos transmission following tag release. To minimise tag drift effects, where the lag between release and first transmission was > 3 days we revert to the most probable geolocation at the time of release to calculate the displacement.

| PTT | Location | Date | FL (cm) | Date | Lon | Lat | Release type | Duration (days) | Displace (km) |
| --- | --- | --- | --- | --- | --- | --- | --- | --- | --- |
| **Yellowfin tuna (*Thunnus albacares*)** | | | | | | | | | |
| 169032 | Young | 2017-05-31 | 135 | 2017-06-01 | -11.858 | -9.765 | Too Deep | 0.8 | 23.7 |
| 169046 | Young | 2017-06-01 | 93 | 2017-06-06 | -12.068 | -9.745 | Premature | 2.3 | 3.0 |
| 169018 | Young | 2018-01-22 | 85 | 2018-01-30 | -12.408 | -9.579 | Premature | 4.5 | 38.2 |
| 169030 | Grattan | 2018-01-24 | 113 | 2018-02-23 | -13.496 | -9.424 | Premature | 27.5 | 80.6 |
| 169037 | Grattan | 2018-01-24 | 115 | 2018-02-15 | -13.672 | -9.720 | Premature | 19.4 | 92.5 |
| 169009 | Grattan | 2018-01-25 | 120 | 2018-03-17 | -12.258 | -9.633 | Floater | 48.2 | 21.4 |
| 169044 | Young | 2018-02-17 | 93 | 2018-05-15 | -12.056 | -9.801 | Pin Broke | 86.6 | 3.2 |
| 169025 | Young | 2018-02-17 | 93 | 2018-03-26 | -12.093 | -9.770 | Pin Broke | 37.4 | 0.0 |
| 169015 | Young | 2018-02-17 | 115 | 2018-02-22 | -13.456 | -9.545 | Too Deep | 5.1 | 71.7 |
| 169013 | Young | 2018-02-17 | 134 | 2018-03-13 | -12.191 | -9.813 | Floater | 20.0 | 32.5 |
| 169007 | Young | 2018-02-17 | 96 | 2018-03-30 | -12.082 | -9.762 | Pin Broke | 41.4 | 0.5 |
| **Bigeye tuna (*Thunnus obesus*)** | | | | | | | | | |
| 165738 | Harris-Stewart | 2017-05-22 | 120 | 2017-07-09 | -17.053 | -8.468 | Pin Broke | 48.7 | 0.0 |
| 165721 | Harris-Stewart | 2017-05-23 | 118 | 2017-06-28 | -19.206 | -7.946 | Premature | 32.7 | 244.0 |
| 165729 | Harris-Stewart | 2017-05-23 | 115 | 2017-12-20 | -17.318 | -8.550 | Premature | 208.0 | 30.5 |
| 165739 | Harris-Stewart | 2017-05-23 | 115 | 2017-10-05 | -17.816 | -8.223 | Floater | 132.0 | 87.5 |
| 169036 | Young | 2017-06-01 | 113 | 2017-07-03 | -12.407 | -9.924 | Premature | 26.8 | 11.2 |
| 169049 | Grattan | 2017-06-02 | 144 | 2017-06-04 | -12.874 | -9.773 | Too Deep | 2.4 | 8.3 |
| 169042 | Young | 2018-01-22 | 120 | 2018-04-11 | -12.210 | -9.470 | Premature | 73.9 | 42.9 |
| 165724 | Young | 2018-01-23 | 129 | 2018-01-26 | -12.480 | -9.588 | Premature | 0.5 | 46.3 |
| 169029 | Grattan | 2018-01-24 | 97 | 2018-02-04 | -12.724 | -9.620 | Floater | 8.5 | 15.0 |
| 169024 | Grattan | 2018-01-24 | 120 | 2018-04-15 | -12.066 | -9.677 | Premature | 77.7 | 9.9 |
| 169011 | Young | 2018-02-17 | 165 | 2018-03-05 | -14.660 | -11.063 | Floater | 12.8 | 248 |

**SUPPLEMENTARY METHODS**

**Bathymetric mapping**

Prior to the current study, limited hydrographic mapping and charting had been conducted on Ascension’s outlying seamounts (none in the case of the Young Seamount). To inform our ecological investigations, high resolution multibeam swath data was therefore collected over the summits of all features down to the 500 m isobath and extended to 1000 m isobath if time permitted. Bathymetric data was collected by the *RRS James Clark Ross* using a hull-mounted 1° x 1° Kongsberg EM122 multibeam echosounder operated using Kongsberg Seafloor Information System (SIS) and Helmsman software. Several local sound velocity profiles derived from expendable bathythermographs (n = 2) and CTD profiles (n = 3) were applied within SIS to calibrate the EM122 data during acquisition. Post-processing was performed in Fledermaus v7.4.0 software in two steps. Firstly, the 3DEditor’s slice-based editing functionality was used to manually flag and reject noisy or erroneous soundings arising, for example, from lost bottom detection or detection of seabed multiples or side lobes. Accepted soundings were then rasterised in the Data Gridding Wizard using a weighted moving average (weight diameter = 3) and 25 m cell size. The final gridded datasets generated can be found in S1 Data.

**Hydroacoustic surveys**

*Data cleaning* – Prior to analysis, acoustic data were cleaned using a series of tools in the Echoview software to exclude noise from the surface (<10m depth from the transducer) and bottom echo (<1 m from the seafloor) and to remove ambient noise, pulse noise and attenuated signals. This noise can be introduced from various sources (e.g. ship propeller, hydraulic winches, bad weather, interference from other electronic instruments). Other sources of noise that could not be identified automatically (e.g. false bottom interference) were eliminated by visual inspection of the echogram and manual editing of the acoustic data. In order to reduce the stochastic variability of the data, the echograms were resampled to a lower resolution (20m × 2m cell) before further steps.

| **Parameter** | **38 kHz** | **120 kHz** |
| --- | --- | --- |
| Sound speed (m/s) | 1543.5 | 1543.5 |
| Absorption coefficient (dB/m) | 0.006315 | 0.047553 |
| Max power (W) | 1000 | 250 |
| Pulse duration (ms) | 1.024 | 1.024 |
| Transducer gain (dB) | 27.77 | 25.11 |
| Sa correction (dB) | 0.01 | -0.05 |
| Alongship 3 dB angle (°) | 6.47 | 5.74 |
| Alongship offset (°) | -0.1 | -0.04 |
| Athwartship 3 dB angle (°) | 6.59 | 5.77 |
| Athwartship offset (°) | 0 | 0.07 |

**Table J**. Echosounder transceiver settings used for hydroacoustic surveys

*Echo classification* – Following Ballon et al., 2011 [1], different echotypes were distinguished using the narrow band frequencies (38 and 120 kHz) with broadband data (70 kHz) used qualitatively to aid in the validation and classification process. Firstly, mean volume backscattering strength (MVBS) at 38 and 120 kHz was summed and the resulting echogram was thresholded in order to separate two broad classes of targets (fish vs plankton). The difference in variability between fish and zooplankton was used to enhance the contrast between both types of organisms. This approach is helpful when there is a high density of gas-bearing plankton that can easily be mistaken for fish if only dB-differencing is used. The threshold value used for diurnal data was empirically set at -135 dB. At night, a more conservative value was used (-133 dB) because the high density of the plankton layer that can be mistakenly classified as fish. Values above the threshold were classified as fish and values below threshold were identified as plankton. A Boolean mask was then created to assign the backscatter to fish and plankton. Fish schools, when present, were detected and assigned to the fish class without going through the thresholding procedure. The fish and plankton categories were further separated into four additional classes (fish with swimbladder, fish without swimbladder, fluid-like plankton, gas-bearing plankton) based on differences in MVBS measured at 120 and 38 kHz (ΔMVBS_120–38_; Table S2).

**Table K**. dB-differencing values used to in taxonomic discrimination

| **Taxon** | **Classes** | **ΔMVBS_120–38_** | **Example** |
| --- | --- | --- | --- |
| Fish | Fish with swimbladder | < 2 |  |
|  | Fish without swimbladder | > 2 |  |
| Plankton | Fluid-like plankton | > 0 | euphausiids, copepods, salps, etc. |
|  | Gas-bearing plankton | < 0 | gelatinous and gas-bearing siphonophores, fish larvae, etc.. |

**Testing for seamount effects**

*Model fitting*

All models were initially fit using the *gam* function in R package *mgcv* using maximum likelihood (ML) smoothing parameter estimation. For models using negative binomial and Tweedie error families, automated functions provided in package *mgcv* were used to select the most appropriate dispersion and power parameters, respectively. Where model checking indicated that more complex structures were necessary, such as random effects with nested autocorrelation structures (for hydroacoustic data), models were refit using the *gamm* function with penalised quasiliklihood (PQL) estimation.

*Model checking*

In a linear modelling context, checks of model misspecification are typically performed by inspecting the residuals for departures from the theoretical quantiles of a normal distribution. However, for many response distributions (e.g. low count data), the residuals are frequently non-normal even under a perfectly fitting model [2]. We therefore used a simulation-based approach to model checking. The method involves repeatedly simulating data from the fitted model, recomputing the residuals and comparing the observed residual distribution to the empirically-derived distribution when the model is correct. Simulated model checks were implemented in the R package *DHARMa* [3] using a range of standard diagnostic plots (QQ-plots, fitted vs. residual) and default tests for non-normality, heteroscedascity, over/underdispersion and zero-inflation. The version of *DHARMa* used had no facility for simulating from negative binomial GAMs so for count data this step was performed in package *mgcViz* [4]. Fitted models were also checked for residual temporal and spatial autocorrelation using implementations of the Durbin-Watson test and Moran’s I test (respectively) provided in *DHARMa*, as well as through manual inspection of lagged autocorrelation plots (*acf* function in R) and spatial semivariograms. Unsurprisingly, strong residual autocorrelation was found in hydroacoustic survey data consisting of continuous measurements along transects and was effectively modelled by including a first order autoregressive (AR1) error process nested within transect (exponential spatial autoregressive in the 2 dimensional case). Significant residual spatial autocorrelation was also detected in the analysis of sea surface chlorophyll satellite data and was again removed by inclusion of an exponential spatial autoregressive term.

*Model simplification*

Model simpliciation was performed by backwards elimination of non-significant terms based on p-values estimated in package mgcv as this provides a consistent method of variable selection for GAMs fit using ML and GAMMs fit using penalised quasilikelihood (PQL) for which Akaike’s Information Criterion (AIC) and other likelihood-based methods of model selection are inappropriate (see S7 Code). The p-values are based on Wald-like tests using the Bayesian variance-covariance matrices of the coeffecients [5] and have been shown to be competitive with alternative methods of model selection (lowest type 1 error rate)[6]. Indeed, for GAMs fit using ML, inferences regarding seamount distance effects were identical whether using stepwise elimination or AIC-based model selection. Where appropriate, the explanatory power of fixed effects was also assessed as the change in the percentage deviance explained when a model with identical smoothing parameters was refit minus the term of interest (deviance explained is not reported for GAMMs fit using PQL).

**Analysis of tuna residency**

Correlated random walk (CRW) simulations were performed using an adaptation of the *simm.crw* function in package *adehabitatLT* for R (see S8 Code). CRWs represent the movement path of the animal as a series of relocations with step lengths and turning angles drawn from appropriate probability density distributions. Initially, we parameterised the CRWs using step length and turning angle distributions derived from the state space model posterior mean tracks. Step length was approximated by a gamma distribution with shape = 1.9 and rate = 0.2, while turning angle was represented by a von Mises distribution with mean = 0 and κ (concentration parameter) = 2.4. For each individual, we then simulated 10,000 CRWs at 12-hourly intervals (i.e. same resolution as the observed tracks) and calculated distance from the tagging seamount at each relative time step. Distance from seamounts at each point in the observed tracks was then compared to the quantiles of the CRW simulations to evaluate the null hypothesis of random dispersal. Where < 5% of simulated tracks ended closer to seamounts than the observed track, this was taken as evidence of site fidelity. Since CRWs parameterised using real tracks risk replicating observed behaviours, we also performed a second set of simulations assuming a constant step length of 1 fork length/second, which represents a reasonable estimate of cruising speed for yellowfin and bigeye tuna [7–9]. To allow more tortuous movement paths, CRWs were simulated at hourly intervals using a von Mises distribution with κ =1 to reduce directional persistence. The results of this parameterisation were qualitatively the same as those based on properties of the observed tracks so only the former are presented.

**REFERENCES**

1. Ballón M, Bertrand A, Lebourges-Dhaussy A, Gutiérrez M, Ayón P, Grados D, et al. Is there enough zooplankton to feed forage fish populations off Peru? An acoustic (positive) answer. Progress in Oceanography. 2011;91: 360–381. doi:10.1016/j.pocean.2011.03.001

2. Wood SN. Inference and computation with generalized additive models and their extensions. TEST. 2020;2020: 307–339.

3. Hartig F. DHARMa: Residual Diagnostics for Hierarchical (Multi-Level / Mixed) Regression Models. 2020. Available: https://CRAN.R-project.org/package=DHARMa

4. Fasiolo M, Nedellec R, Goude Y, Wood SN. Scalable Visualization Methods for Modern Generalized Additive Models. Journal of Computational and Graphical Statistics. 2020;29: 78–86. doi:10.1080/10618600.2019.1629942

5. Wood SN. On p-values for smooth components of an extended generalized additive model. Biometrika. 2013;100: 221–228. doi:10.1093/biomet/ass048

6. Marra G, Wood SN. Practical variable selection for generalized additive models. Computational Statistics & Data Analysis. 2011;55: 2372–2387. doi:10.1016/j.csda.2011.02.004

7. Yuen HSH. Swimming Speeds of Yellowfin and Skipjack Tuna. Transactions of the American Fisheries Society. 1966;95: 203–209. doi:10.1577/1548-8659(1966)95[203:SSOYAS]2.0.CO;2

8. Dagorn L, Bach P, Josse E. Movement patterns of large bigeye tuna (Thunnus obesus) in the open ocean, determined using ultrasonic telemetry. Marine Biology. 2000;136: 361–371. doi:10.1007/s002270050694

9. B. A. Block, J.E. Keen, B. Castillo, H. Dewar, E.V. Freund, D.J. Marcinek, et al. Environmental preferences of yellowfin tuna (Thunnus albacares) at the northern extent of its range.pdf. Marine Biology; 1997.
