# Supplementary figures and images for: Shallow seamounts are “oases” and activity hubs for pelagic predators in a large-scale marine reserve
Source: PLoS Biol. 2025 Feb 4;23(2):e3003016. doi: 10.1371/journal.pbio.3003016 (PMC11828362; doi:10.1371/journal.pbio.3003016)

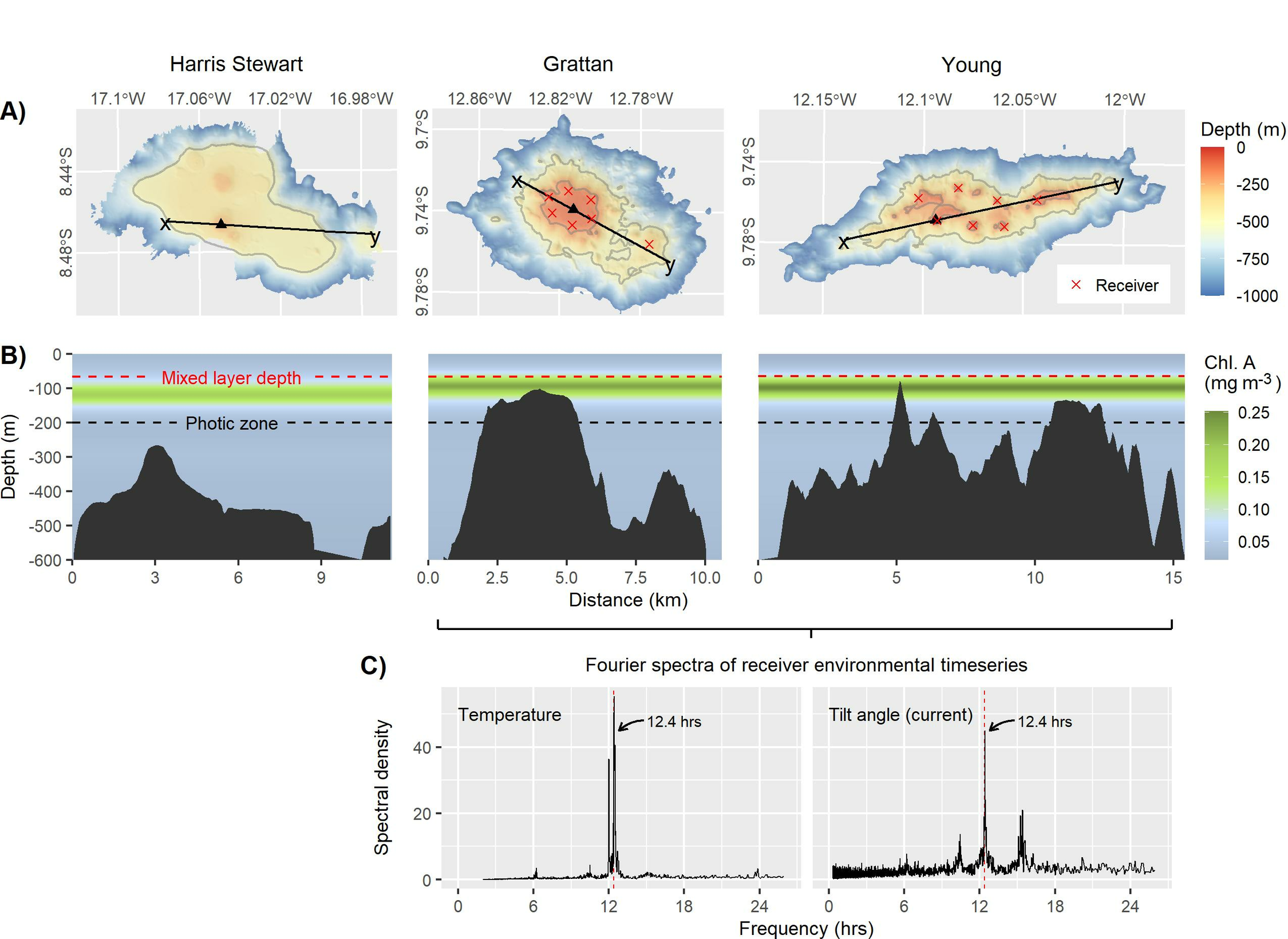

Supplement: S1 Fig — (A) High-resolution (25 m) multibeam bathymetry of the Harris Stewart, Grattan and Young seamounts. Triangle markers denote the shallowest point of each feature. (B) Bathymetric profiles along transect lines (x → y) in (A) overlaid on mean water column chlorophyll A concentration in 5 m depth increments calculated from all CTD deployments made over each feature (May–June 2017). The mean depth of the surface mixed layer (65–67 m) in CTD deployments and the approximate limit of the photic zone (200 m) are also shown for reference. (C) Periodograms of 595-day temperature and current strength time series recorded by 14 acoustic telemetry receivers deployed on the summits of the Grattan and Young seamounts (positions marked in (A)). Plots show the mean spectral density averaged across all receivers for the 0–26 h frequency range. Note the dominant 12.4-h periodicity in both series corresponding to the frequency of the principle semi-diurnal lunar tide. The data underlying this figure can be found in S1 Data (A) and S5 Data (B and C). (TIF) [file pbio.3003016.s002.tif]

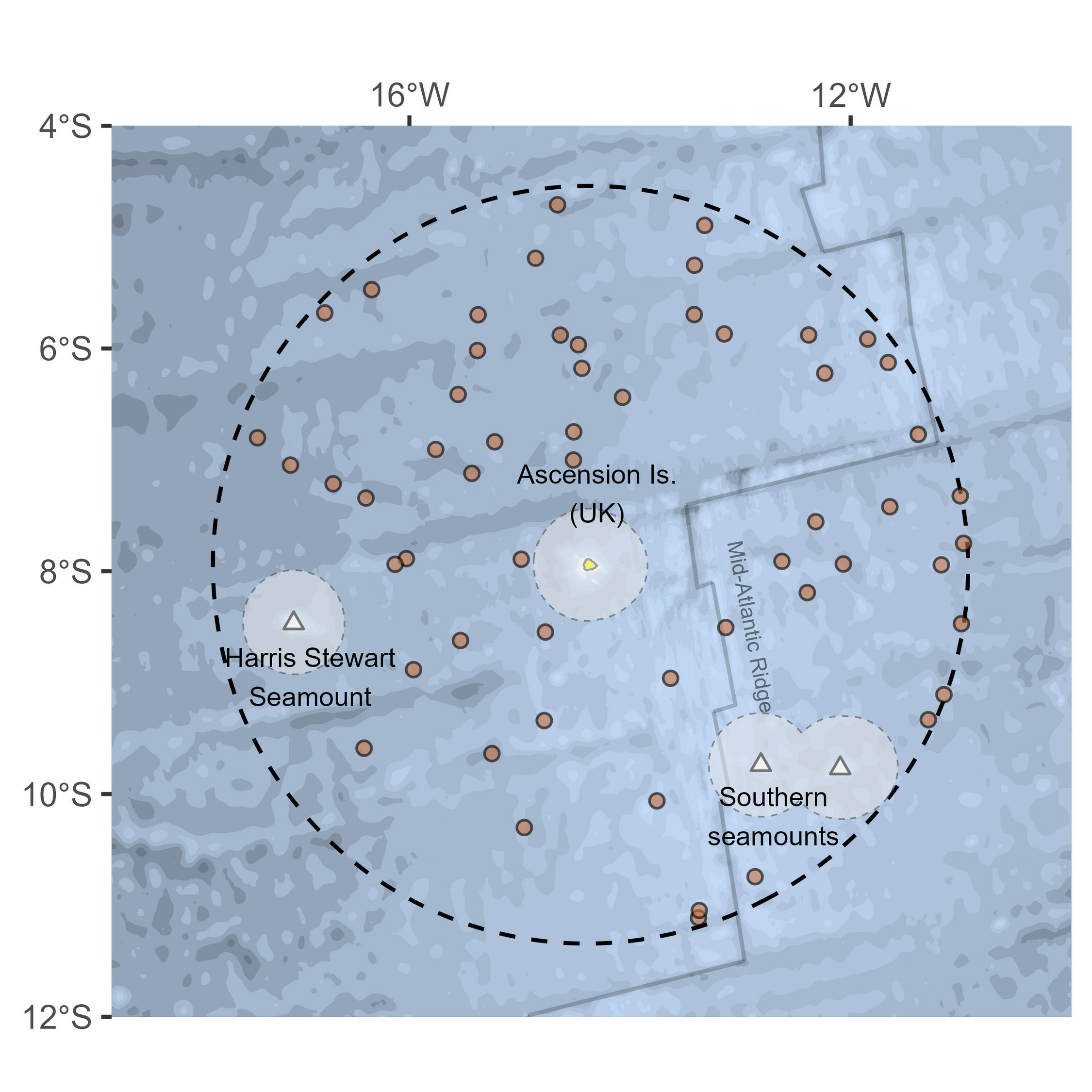

Supplement: S2 Fig — The 50 km feature buffers are marked in white. Data from these surveys was used to provide additional estimates of baseline abundances of pelagic predators in addition to those derived from radial sampling around seamounts (see Table B in S1 Text). The data underlying this figure can be found in S2 Data. (TIF) [file pbio.3003016.s003.tif]

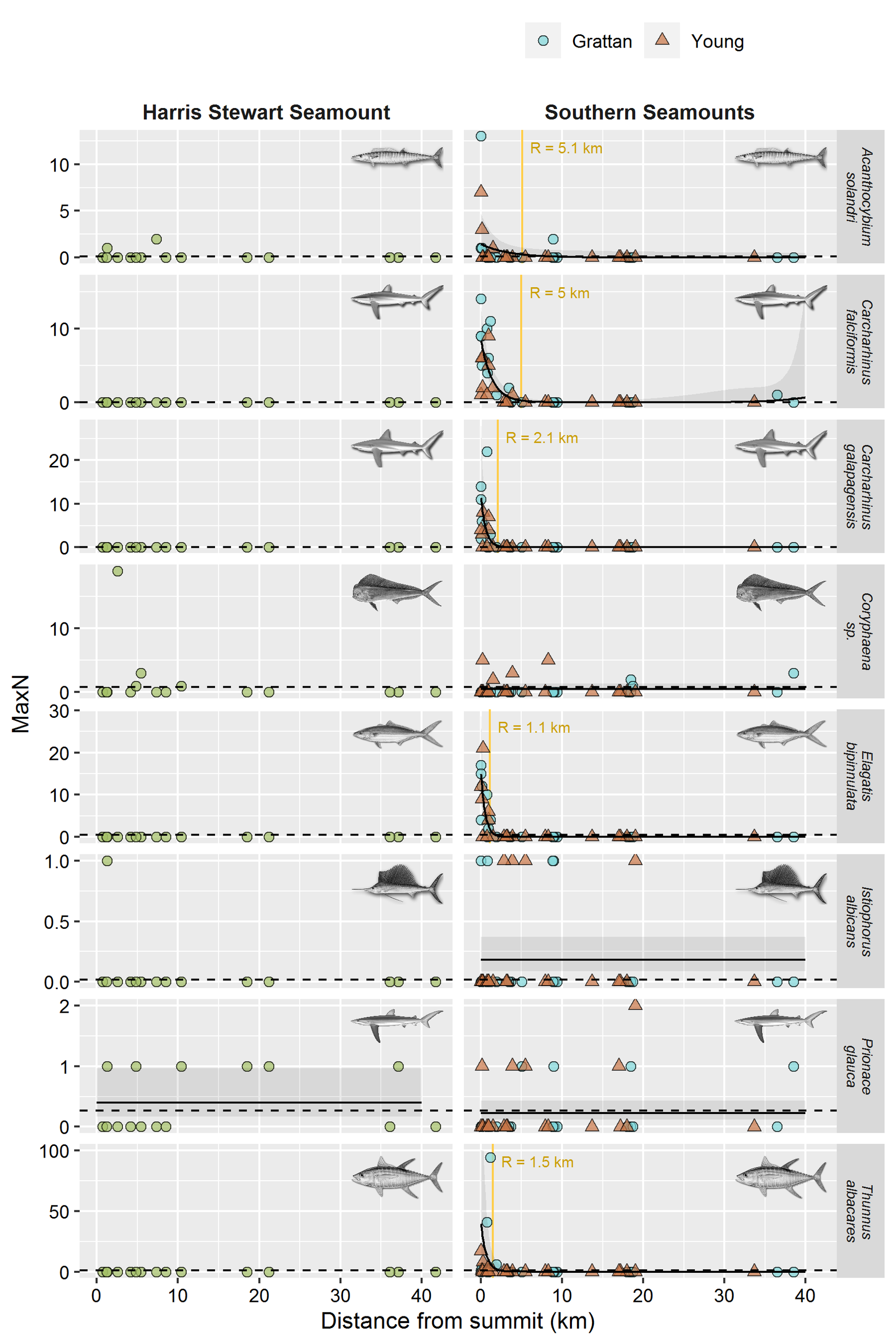

Supplement: S3 Fig — Relative abundance is expressed as the maximum number of individuals of a given species observed in a single video frame (MaxN). Only data from species observed in >5 surveys are presented. Explanations of other plotting elements follow Fig 2. The data underlying this figure can be found in S2 Data. Illustrations: Marc Dando (sharks) and Diane Rome Peebles (fish). (TIF) [file pbio.3003016.s004.tif]

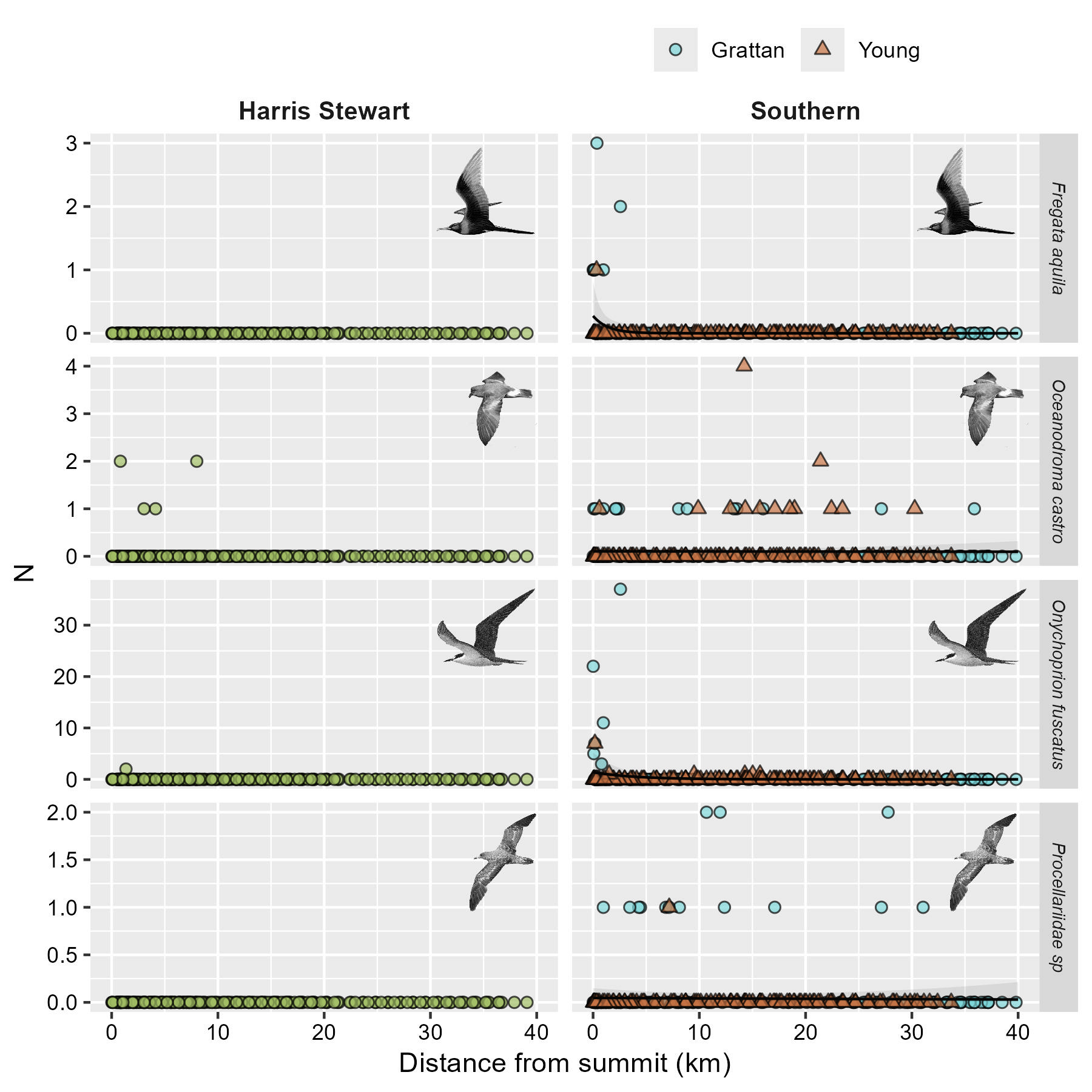

Supplement: S4 Fig — Relative abundance is expressed as the number of individuals observed in a 300 m belt transect centred on the vessel per 5 min sampling interval. Only data from species observed in >5 sampling intervals are presented. Solid trend lines and shaded envelopes are fitted smooths and associated 95% CIs from negative binomial GAMMs with a random effect of transect id. Illustrations: F. aquila and O. fuscatus, Peter Harrison; O. Castro and Procellaridae sourced via Wikimedia under a Creative Commons license. The data underlying this figure can be found in S3 Data. (TIF) [file pbio.3003016.s005.tif]

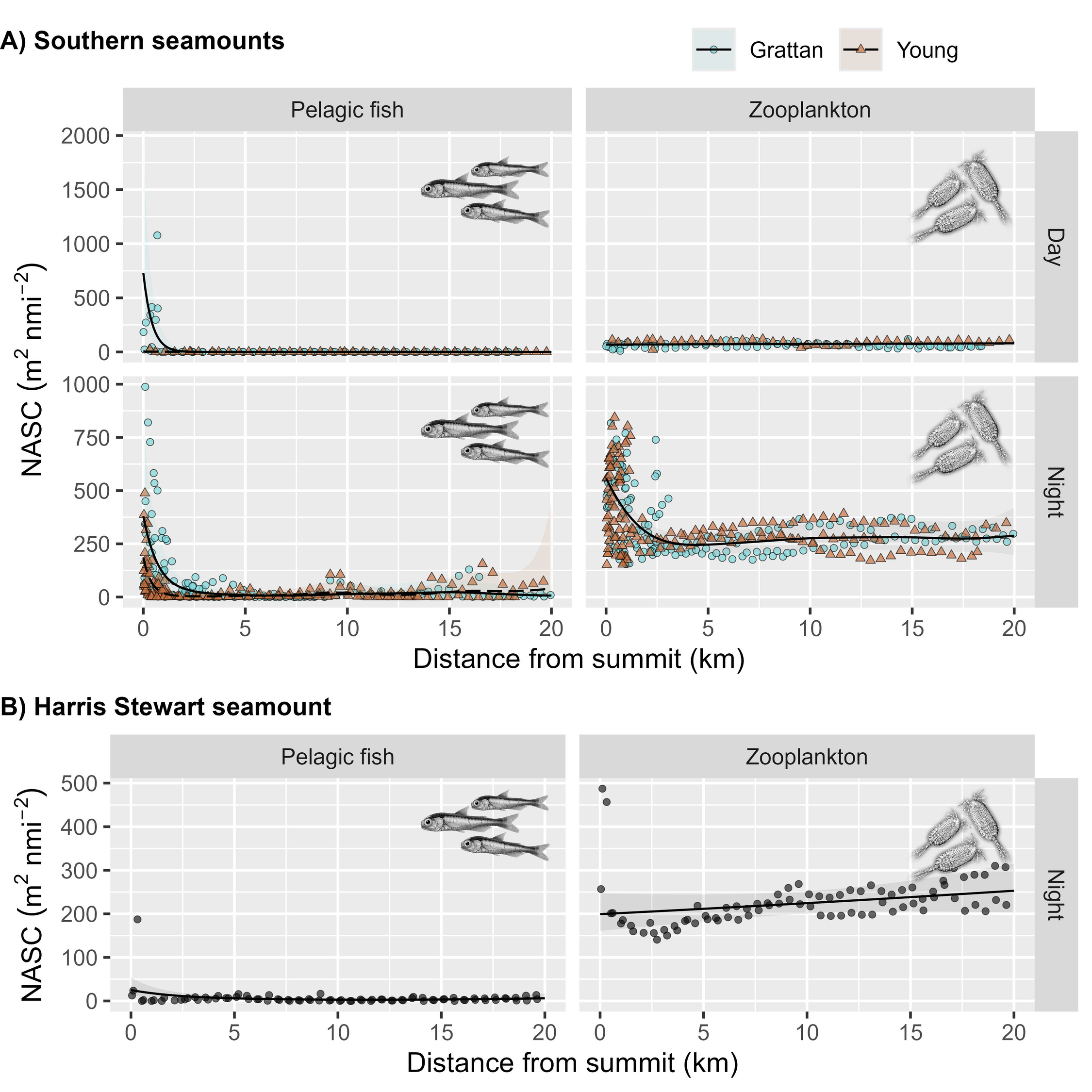

Supplement: S5 Fig — Plotted points are total water column NASC from 0–300 m depth, integrated over 500 m distance sampling units and trend lines are predicted means from fitted GAMs and their associated 95% confidence intervals (see Table E in S1 Text). Fitted smooths were initially allowed to vary for the 2 Southern Seamounts but were only retained for pelagic fish where trends were significantly non-overlapping. For zooplankton, a single global smoother is presented. The data underlying this figure can be found in S4 Data. Illustrations: Creative Commons via Wikimedia. (TIF) [file pbio.3003016.s006.tif]

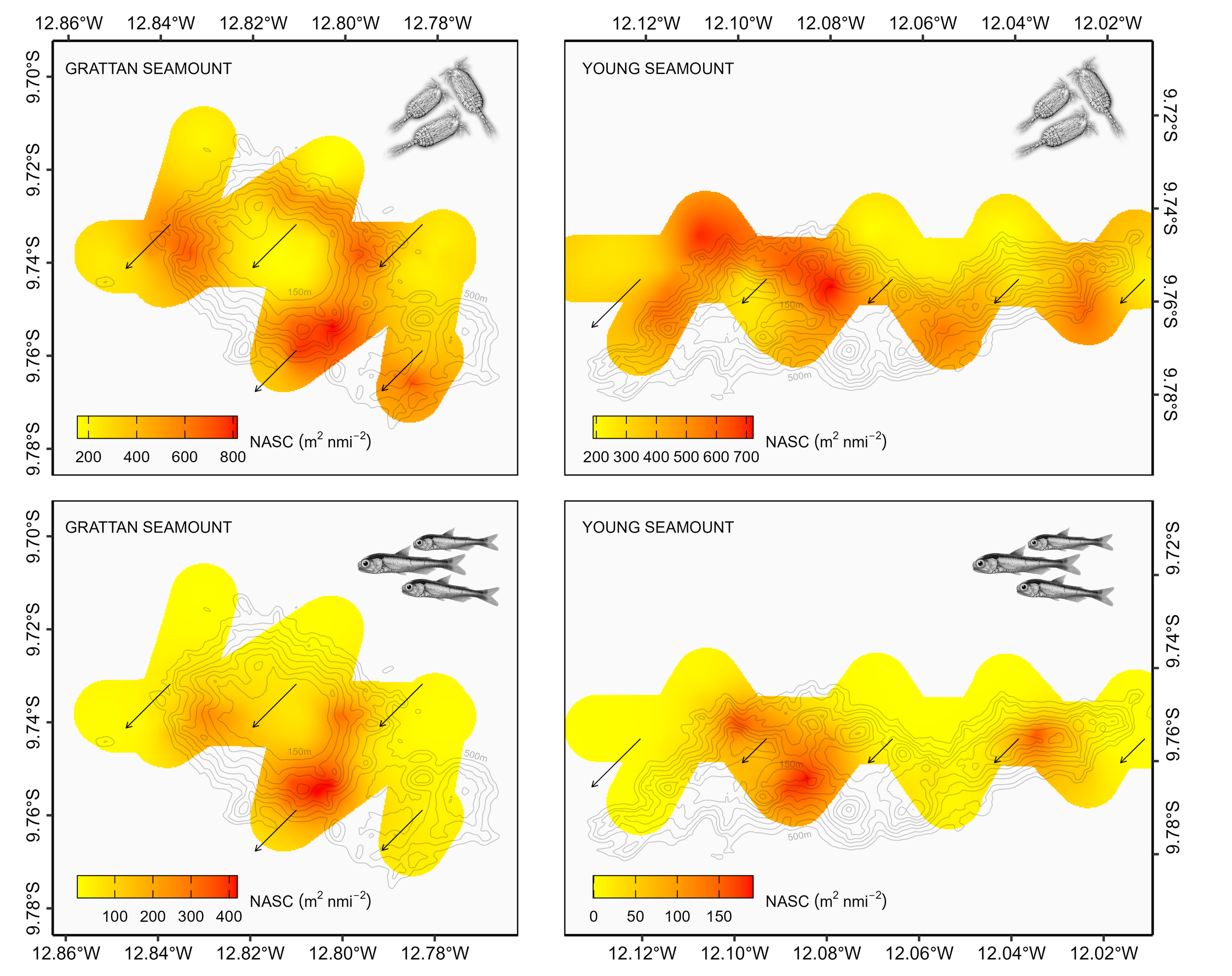

Supplement: S6 Fig — Heatmaps were derived using ordinary kriging of total water column (0–300 m) nautical area scatterning coefficients (NASCs) integrated over 500 m distance sampling units. To avoid over-interpolation, kriging was limited to an area within 800 m of the survey transect. Arrow vectors in each plot represent the mean geostropic current flows over each seamount during hydroacoustic surveys based on time-matched satellite altimery data (source: Copernicus Marine Service GlobCurrent, dataset MULTOBS_GLO_PHY_MYNRT_015_003). The data underying this figure can be found in S4 Data. Illustrations: Creative Commons via Wikimedia. (TIF) [file pbio.3003016.s007.tif]

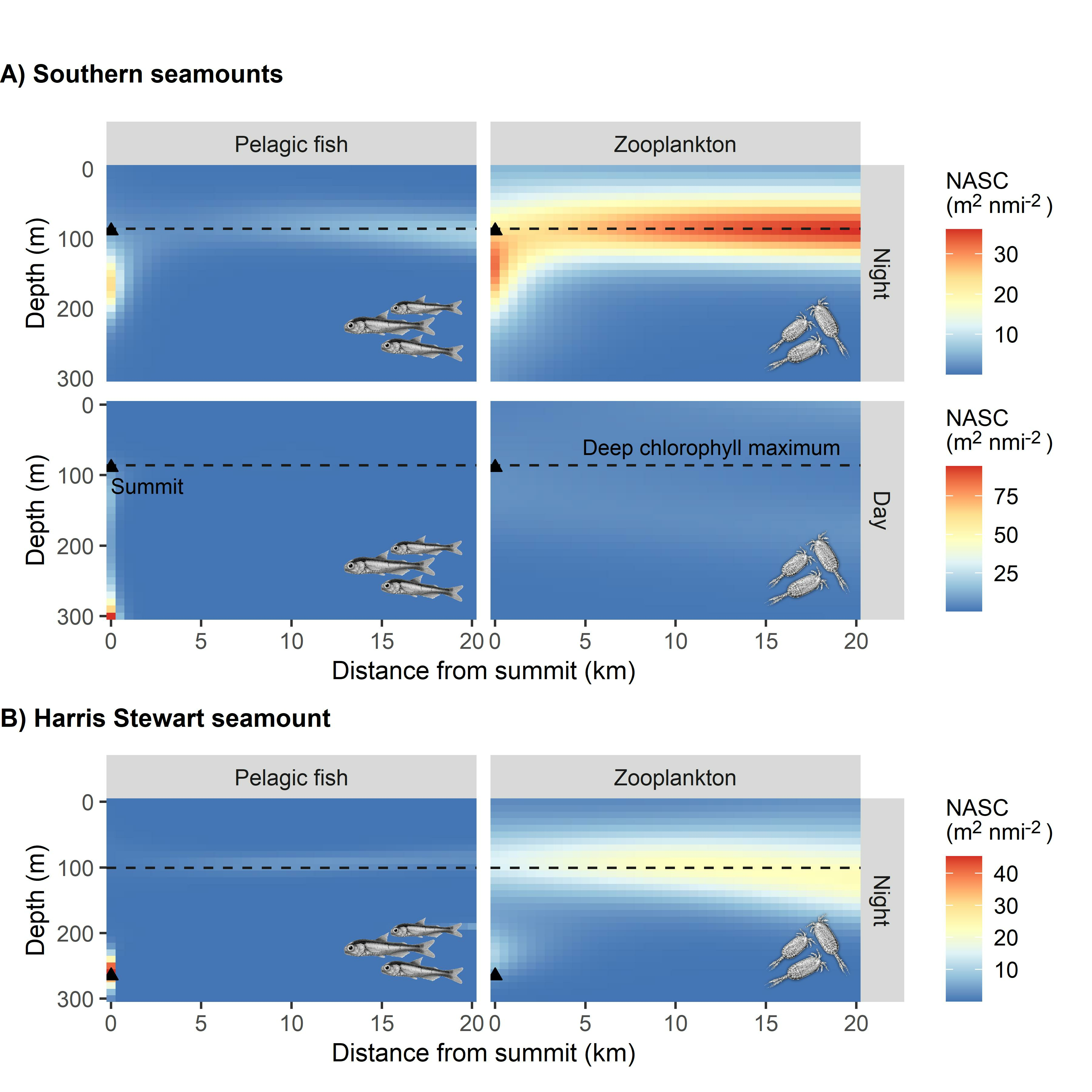

Supplement: S7 Fig — Heatmaps were derived using ordinary kriging of total water column (0–300 m) nautical area scatterning coefficients (NASCs) integrated over 500 m distance sampling units. To avoid over-interpolation, kriging was limited to an area within 800 m of the survey transect. Arrow vectors in each plot represent the mean geostropic current flows over each seamount during hydroacoustic surveys based on time-matched satellite altimery data (source: Copernicus Marine Service GlobCurrent, dataset MULTOBS_GLO_PHY_MYNRT_015_003). The data underying this figure can be found in S4 Data. Illustrations: Creative Commons via Wikimedia. (TIF) [file pbio.3003016.s008.tif]

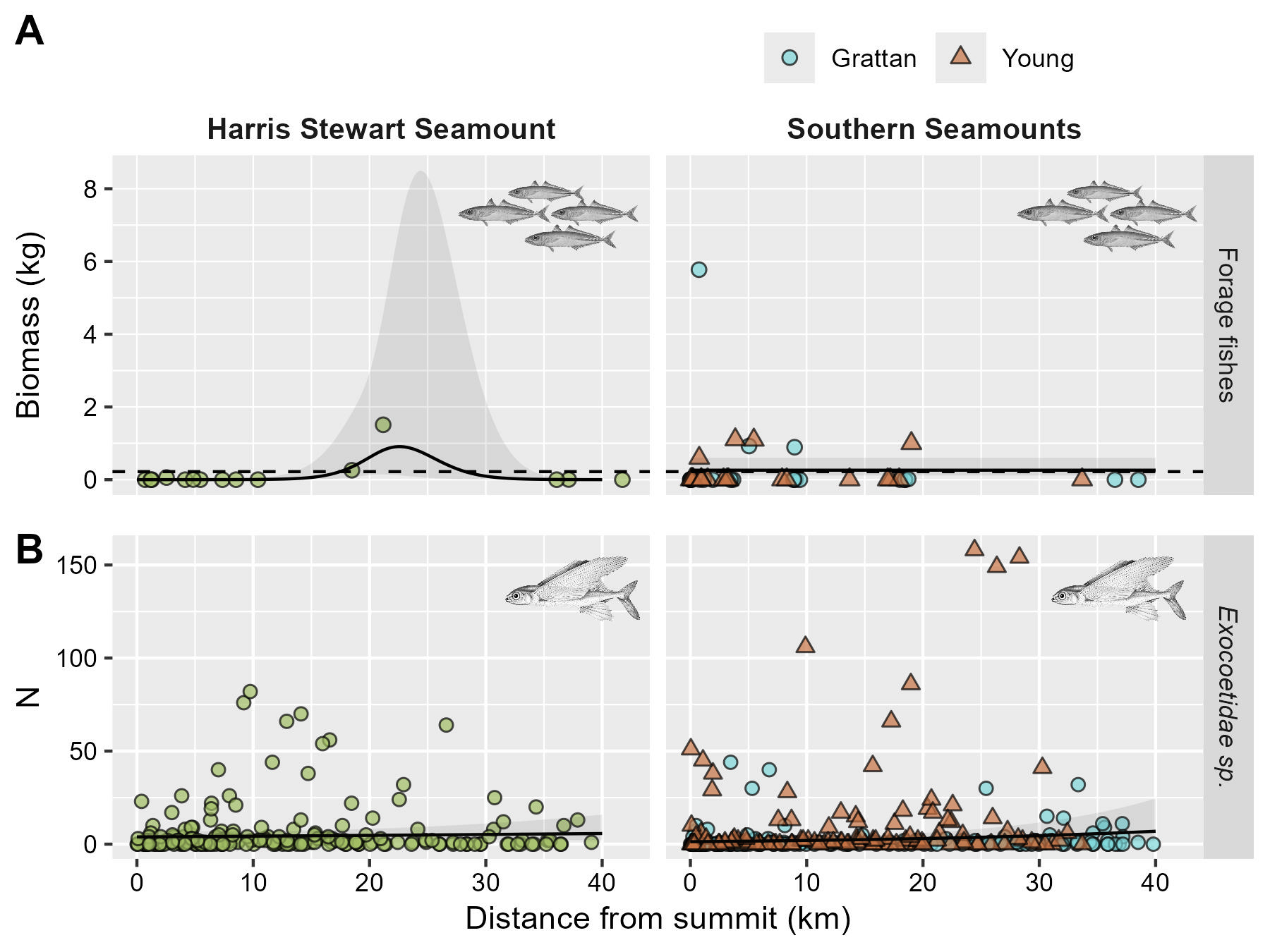

Supplement: S8 Fig — (A) Relative biomass of epipelgic forage fish observed in shallow (10 m) pelagic BRUV deployments. Biomass is expressed as the maximum biomass of forage fish (see Table B in S1 Text) observed in a single video frame, based on photogrammetric fork length measurements of individual animals converted using species-specific length–weight relationships (see Methods). Broken lines represent regional oceanic baselines from a reference set of 56 BRUVs surveys conducted >50 km from any seamount or island over a similar period (S2 Fig). (B) Relative abundance of flying fish (Exocoetidae sp.) observed in vessel-based visual transects. Abundance (N) is expressed as the number of individuals observed in a 300 m belt transect centred on the vessel per 5 min sampling interval. Solid trend lines and shaded envelopes are fitted smooths and associated 95% confidence intervals from Tweedie GAMs for biomass and negative binomial GAMMs with a random effect of transect id for visual transects. The data underlying this figure can be found in S2 Data (A) and S3 Data (B). Illustrations: Creative Commons via Wikimedia. (TIF) [file pbio.3003016.s009.tif]

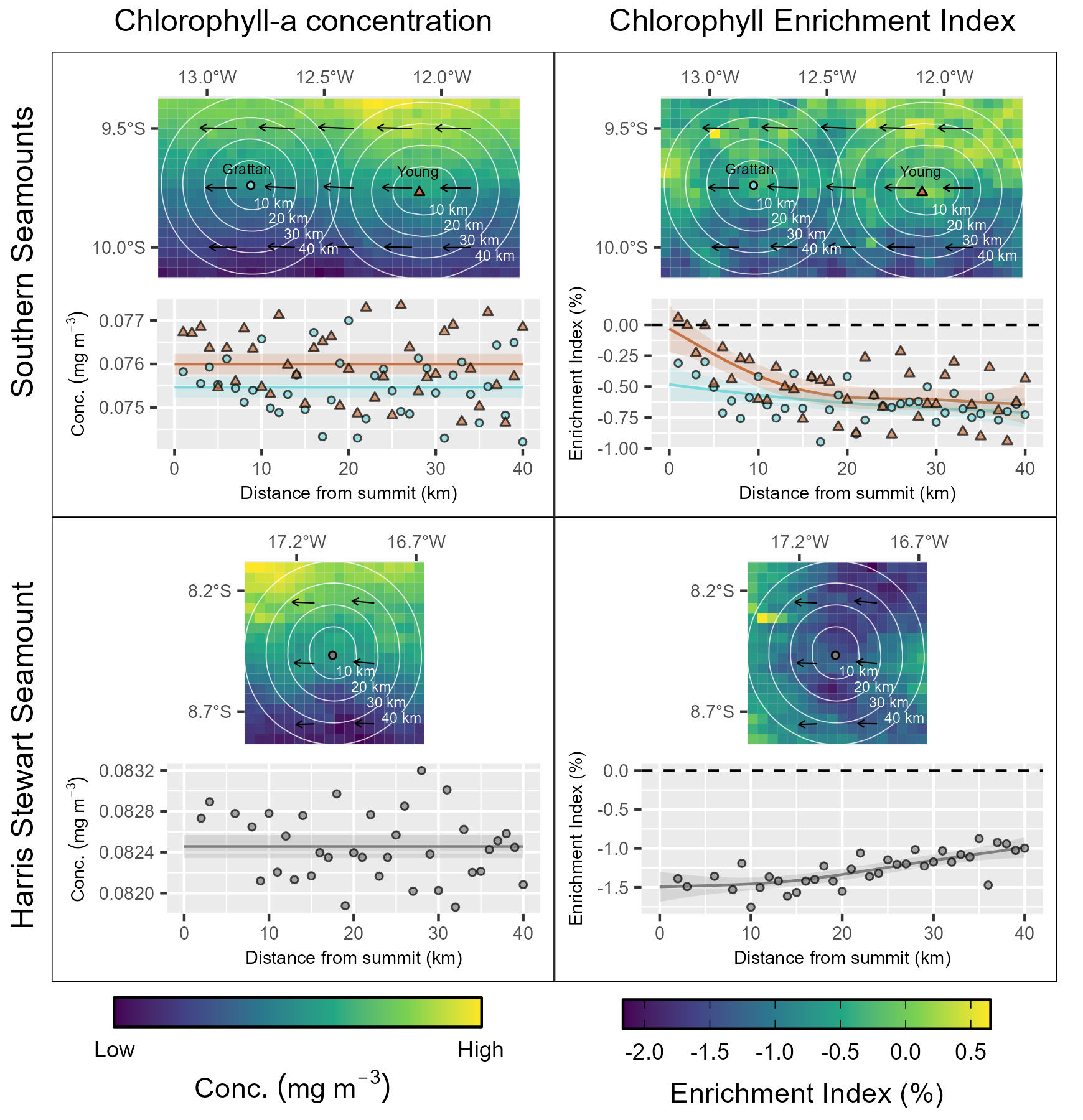

Supplement: S9 Fig — CEIs are percentage deviations from a moving average of all cells located within 30–90 km of the focal cell and help strengthen any local seamount signal by removing mesoscale gradients (see Methods). In each map, arrow vectors show the average current strength and direction across the study period based on satellite altimetry data (source: Copernicus Marine Service, dataset MULTIOBS_GLO_PHY_REP_015_004). Plots below each map show how mean chlorophyll concentrations and enrichment indices vary as a function of distance from the nearest seamount summit. In each case, plotted points are the mean cell values calculated in 1 km increments radiating out from the summit and trend lines are predictions from fitted GAMs and associated 95% confidence intervals (see Table G in S1 Text). The data underlying this figure can be found in S5 Data. (TIF) [file pbio.3003016.s010.tif]

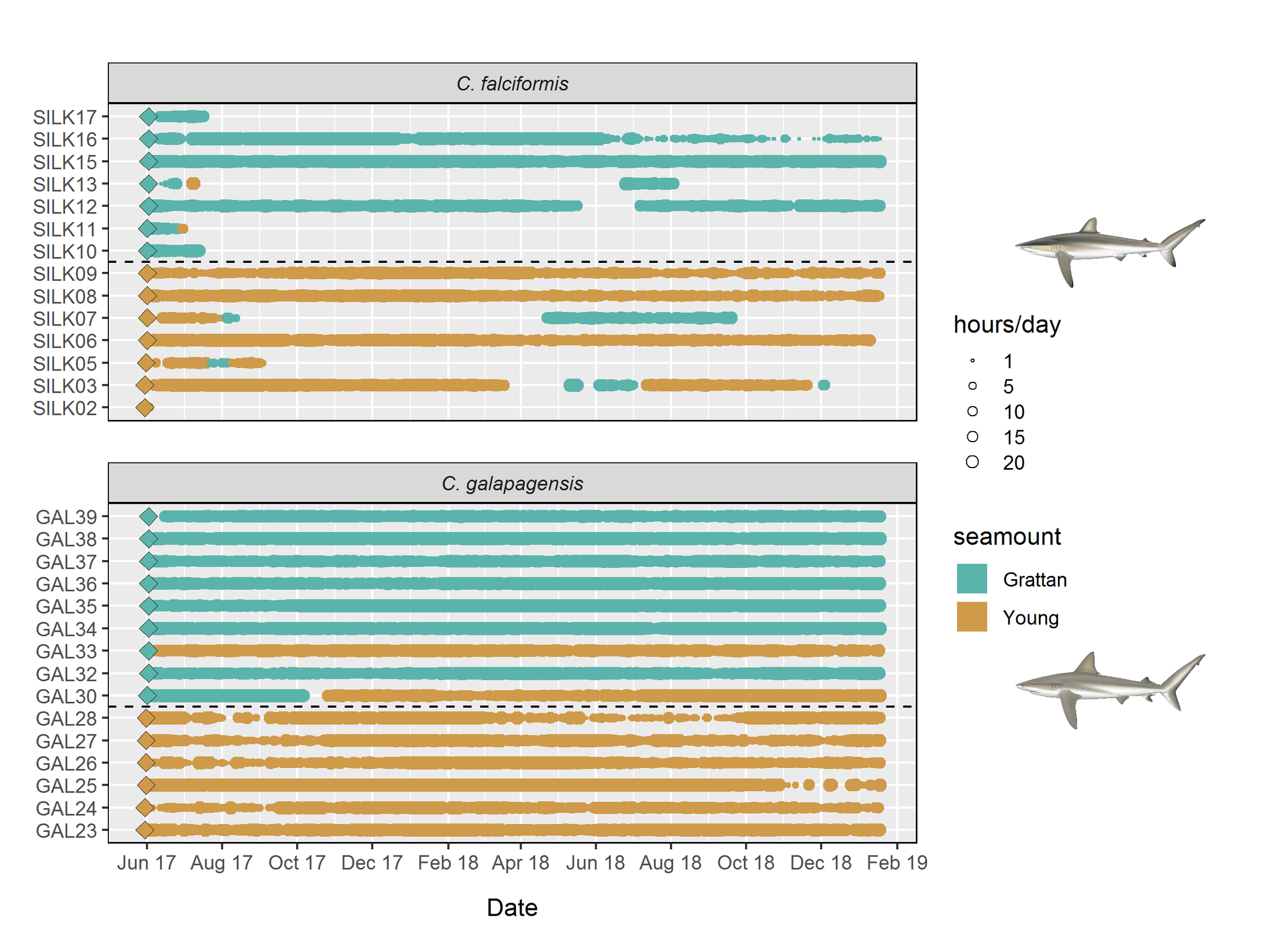

Supplement: S10 Fig — Symbols are shaded according to the seamount on which detections occurred and are scaled according to the number of hourly bins on each day in which an animal was detected at least once on summit receiver arrays (S1 Fig). Note the periodic movements between seamounts observed in some individuals. The data underlying this figure can be found in S6 Data. Illustrations: Marc Dando. (TIF) [file pbio.3003016.s011.tif]
